# Current Approaches to Aflatoxin B1 Control in Food and Feed Safety: Detection, Inhibition, and Mitigation

**DOI:** 10.3390/ijms26136534

**Published:** 2025-07-07

**Authors:** Katarzyna Kępka-Borkowska, Katarzyna Chałaśkiewicz, Magdalena Ogłuszka, Mateusz Borkowski, Adam Lepczyński, Chandra Shekhar Pareek, Rafał Radosław Starzyński, Elżbieta Lichwiarska, Sharmin Sultana, Garima Kalra, Nihal Purohit, Barbara Gralak, Ewa Poławska, Mariusz Pierzchała

**Affiliations:** 1 Department of Genomics and Biodiversity, Institute of Genetics and Animal Biotechnology of the Polish Academy of Sciences, Jastrzębiec, Postępu St. 36A, 05-552 Magdalenka, Poland; k.kepka@igbzpan.pl (K.K.-B.); k.chalaskiewicz@igbzpan.pl (K.C.); m.ogluszka@igbzpan.pl (M.O.); b.gralak@igbzpan.pl (B.G.); m.pierzchala@igbzpan.pl (M.P.); 2 Department of Commercialization and Technology Transfer, Industrial Chemistry Institute, Łukasiewicz Research Network, Warsaw, Rydygiera St. 8, 01-793 Warsaw, Poland; mateusz.borkowski@ichp.lukasiewicz.gov.pl; 3 Department of Physiology, Cytobiology and Proteomics, West Pomeranian University of Technology, Szczecin, K. Janickiego St. 29, 71-270 Szczecin, Poland; adam.lepczynski@zut.edu.pl (A.L.); elzbieta_lichwiarska@zut.edu.pl (E.L.); 4 Institute of Veterinary Medicine, Department of Infectious, Invasive Diseases and Veterinary Administration, Faculty of Biological and Veterinary Sciences, Nicolaus Copernicus University, Toruń, Lwowska St. 1, 87-100 Toruń, Poland; pareekcs@umk.pl (C.S.P.); ssharna21@gmail.com (S.S.); vetgarimakalra@gmail.com (G.K.); purohitnihal621@gmail.com (N.P.); 5 Department of Molecular Biology, Institute of Genetics and Animal Biotechnology of the Polish Academy of Sciences, Jastrzębiec, Postępu St. 36A, 05-552 Magdalenka, Poland; r.starzynski@igbzpan.pl

**Keywords:** aflatoxins, mycotoxins, food safety

## Abstract

Aflatoxins, toxic secondary metabolites produced primarily by *Aspergillus flavus* and *Aspergillus parasiticus*, pose a significant global health concern due to their frequent presence in crops, food, and feed—especially under climate change conditions. This review addresses the growing threat of aflatoxins by analyzing recent advances in detection and mitigation. A comprehensive literature review was conducted, focusing on bioremediation, physical and chemical detoxification, and fungal growth inhibition strategies. The occurrence of aflatoxins in water systems was also examined, along with current detection techniques, removal processes, and regulatory frameworks. Emerging technologies such as molecular diagnostics, immunoassays, biosensors, and chromatographic methods are discussed for their potential to improve monitoring and control. Key findings highlight the increasing efficacy of integrative approaches combining biological and technological solutions and the potential of AI-based tools and portable devices for on-site detection. Intelligent packaging and transgenic crops are also explored for their role in minimizing contamination at the source. Overall, this review emphasizes the importance of continued interdisciplinary research and the development of sustainable, adaptive strategies to mitigate aflatoxin risks, thereby supporting food safety and public health in the face of environmental challenges.

## 1. Introduction

*Aspergillus flavus* and *Aspergillus parasiticus* are the primary fungal species responsible for the production of aflatoxins. The four main types of aflatoxins are AFB1 (aflatoxin B1), AFB2 (aflatoxin B2), AFG1 (aflatoxin G1), and AFG2 (aflatoxin G2), ranked from most to least toxic as follows: AFB1, AFG1, AFB2, and AFG2 [1,2]. Most *A. flavus* isolates secrete aflatoxins B1 and B2, while some strains can also produce aflatoxin types G1 and G2. In contrast, *A. parasiticus* synthesizes all four types of aflatoxins [3]. These mycotoxins are highly hazardous, as they have been classified by the International Agency for Research on Cancer as group 1 human carcinogens [4]. It has been shown that aflatoxins exhibit strong carcinogenic, hepatotoxic, nephrotoxic, teratogenic, and mutagenic properties, and they can also cause autoimmune issues in animals and humans. The primary route of exposure to aflatoxins is through oral ingestion [2,5,6,7,8].

Aflatoxin B1 is one of the most potent naturally occurring toxins, with a toxicity up to 68 times greater than arsenic [9]. In mammals, AFB1 is metabolically converted to AFM1, which can subsequently contaminate milk and dairy products [10]. Aflatoxin contamination of food can occur at various stages, including crop cultivation, seed storage, animal feed production, and processing of plant and animal products. Aflatoxins have also been detected in meat, milk, water, and soil [6]. It is estimated that aflatoxins are present in one-quarter of the global food supply [3,7]. Acute aflatoxin poisoning can cause symptoms such as fever, abdominal pain, vomiting, edema, and liver failure [11]. Chronic exposure may lead to immune system suppression, hepatocellular carcinoma, birth defects, and neurological damage [12]. Although the WHO and FAO have established guidelines on maximum allowable levels of aflatoxins [13], climate change is expected to exacerbate the problem [14].

Therefore, the development of new and effective methods for detecting and eliminating aflatoxins is crucial. Advances in analytical technologies and information systems have significantly improved the speed, accuracy, and accessibility of detection techniques [15]. However, many current decontamination approaches have shown limited effectiveness or face challenges such as high costs, and continuous chemical use, and potential reductions in the nutritional quality of food [16].

Various strategies for controlling aflatoxin levels can be broadly categorized into two main groups: (1) prevention of fungal contamination and inhibition of fungal growth, and (2) detoxification of already contaminated products [17,18]. Disinfection techniques involving physical, chemical, and biological agents are employed to reduce or eliminate aflatoxins from food, thereby minimizing associated health risks [19].

This introduction highlights the critical importance of aflatoxin B1 as a persistent food safety threat. While numerous reports have documented its toxicological profile, there remains an urgent need to contextualize aflatoxin-related risks within emerging challenges such as climate change and the globalization of food systems. This review examines current methods for aflatoxin detection and various inhibition strategies, including bioremediation agents, physical agents, and both botanical and non-botanical chemical compounds with antifungal and antitoxic effects. Additionally, it explores technological advancements in detection and mitigation, such as artificial intelligence, machine learning, intelligent packaging, and active packaging, based on studies published between 2014 and 2025.

## 2. Regulations on Aflatoxins in Animal Feed: An Analysis of Key Producing Countries

An important consideration is the variation in permissible aflatoxin concentration limits in animal feed, which has significant implications for human health, given that people consume animal-derived products. In animals that ingest aflatoxin-contaminated feed, toxic metabolites formed through biotransformation can accumulate in tissues, pass into eggs, and be excreted in milk [20]. In the European Union, aflatoxin levels are regulated under Commission Regulation (EU) 2023/915 for food and Regulation (EC) 1881/2006 for animal feed. The established maximum levels of aflatoxin B1 (assuming a moisture content of 12%) are as follows:

- Feed materials: 20 μg/kg.
- Complementary and complete feed mixtures: 10 μg/kg.
- Feed mixtures for dairy cattle and calves, dairy sheep and lambs, dairy goats and kids, piglets, and young poultry: 5 μg/kg.
- General feed mixtures for other categories not specified above: 20 μg/kg [21].

In comparison, the standards set by the Missouri Department of Agriculture are summarized in Table 1.

On a global scale, studies indicate that regions such as Africa, the Middle East, South Asia, and parts of Southern Europe typically exhibit high concentrations of aflatoxins. China, one of the countries most affected by mycotoxin contamination, has tightened its regulations in recent years concerning permissible levels of mycotoxins in animal feed and raw materials [23]. These regulatory limits are differentiated based on animal age, species, intended use, and type of feed component (see Table 2). Notably, feed samples collected in China in 2021 demonstrate a relatively low incidence of aflatoxin contamination [22].

Climatic conditions in Brazil—one of the world’s leading agricultural producers—are highly conducive to the proliferation of toxigenic fungal species and the subsequent production of mycotoxins [20]. In 2011, the Brazilian Ministry of Agriculture issued an executive order recommending a maximum allowable concentration of aflatoxins B1, B2, G1, and G2 in raw materials used for feed production of 50 µg/kg. For peanuts and corn, the limit is set at 20 µg/kg [24]. However, studies have shown that these standards are frequently exceeded, underscoring the urgent need for effective measures to prevent and remove aflatoxin contamination [20].

In India, the Bureau of Indian Standards (BIS) has set a uniform maximum limit of 20 µg/kg for aflatoxin B1 across all types of animal feed. Like Brazil, India experiences climatic conditions favorable to mold proliferation. Recent research highlights the need for India and other developing nations to adopt sustainable preventive strategies for managing crops both pre- and post-harvest. These strategies should include continuous monitoring and targeted interventions during storage and processing (e.g., in corn silage) to reduce aflatoxin levels [25]. Implementing such approaches—alongside stricter regulatory thresholds—would mitigate economic losses and enhance the safety of animal-derived food products for consumers.

Regulatory thresholds for aflatoxins vary significantly across regions, reflecting not only climatic differences but also disparities in analytical capabilities and food safety priorities. These inconsistencies can result in uneven levels of consumer protection worldwide. The harmonization of aflatoxin limits, particularly for trade-sensitive commodities, remains a critical policy objective. Moreover, future regulations may need to evolve dynamically in response to shifting aflatoxin prevalence driven by climate change.

## 3. Aflatoxin Detection

**Figure 1:**
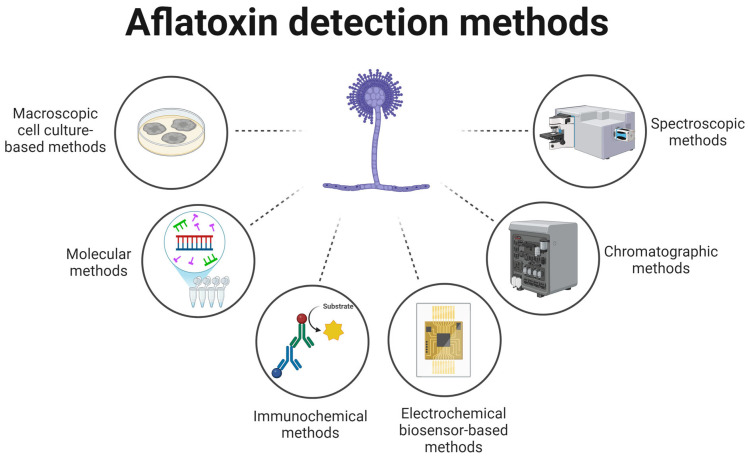
Types of AFB1 detection methods.

The detection of aflatoxins is critical to ensuring the safety of both food and feed. Detection methods are commonly categorized into six main groups: macroscopic culture-based techniques, molecular assays, immunochemical approaches, electrochemical biosensors, chromatographic systems, and spectroscopic tools (Figure 1).

### 3.1. Macroscopic Culture-Based Methods

Macroscopic culture methods, employing media such as coconut agar medium (CAM), coconut milk agar (CMA), yeast extract sucrose (YES), and aflatoxin-producing ability (APA) medium, enable the detection of toxigenic *Aspergillus* strains by observing blue fluorescence under UV light [26,27,28]. Results are typically visible within 32 to 120 h. Palm kernel substrates may also differentiate aflatoxigenic isolates through the appearance of yellow pigmentation [29].

These methods are simple and cost-effective; however, they require microbiological expertise. Due to their time-consuming nature, they are more suitable for laboratory confirmation than for high-throughput screening in routine inspections.

### 3.2. Molecular Methods

Molecular assays target aflatoxin biosynthesis genes such as *aflD, aflM, aflR*, and *aflJ*, most commonly utilizing conventional or real-time PCR [30,31,32]. Table 3 summarizes the application of molecular techniques in detecting contaminated food.

Although highly sensitive, these methods detect the presence of aflatoxigenic fungi rather than the aflatoxins themselves. They require specialized equipment and trained personnel, resulting in relatively high operational costs.

### 3.3. Immunochemical Methods

Immunochemical assays, particularly enzyme-linked immunosorbent assay (ELISA) and lateral flow devices, are widely applied for the rapid detection of aflatoxins in various food matrices such as milk, edible oils, peanuts, cereals, and spices [35,36,37]. Examples of these applications are provided in Table 4.

These methods are accessible, easy to use, and cost-effective, enabling the simultaneous detection of multiple mycotoxins. However, they may be affected by matrix interferences, exhibit limited sensitivity, and often provide only semiquantitative results [38].

**Table 4:** Immunochemical methods used to detect aflatoxins in food.

Method	Detecting Matrix	Detected Aflatoxin	Limit of Detection for Aflatoxins [μg/kg]	References
Rapid immunochromatographic strip	Monoclonal antibody-gold nanoparticles (mAb-AuNP)	AFB1	1.0	[39]
Monoclonal antibody-based fluorescent microsphere immunochromatographic test strip	Fluorescent microspheres–mAb	AFM1	4.4	[40]
One-step immunochromatographic assay	mAb-AuNP	AFM1	0.05 (EU) 0.5 (others)	[41]
Quantum dot nanobead-based multiplexed immunochromatographic assay	Quantum dot nanobead with antibody	AFB1	0.00165	[42]
Gold nanoparticle-based conjugated AFB1 antifungal strips	AuNPs conjugated with AFB1 antibody and bovine serum albumin	AFB1	10	[43]
Nanoparticle-based competitive magnetic immunodetection	Biotinylated mAb, magnetic particles functionalized with streptavidin	AFB1	1.1	[44]
Two-analyte immunochromatographic strip	Protein conjugates (AFM1-OVA (aflatoxin M1–ovalbumin conjugate) and chloramphenicol-ovalbumin) and goat anti-rabbit IgG (immunoglobulin G)	AFM1	0.1	[45]
Immunochromatographic test	Antigen-modified Fe_2_O_3_ nanoprobes	AFB1	0.0125	[36]
Pressure/colorimetric dual-readout immunochromatographic test strip	Dendritic platinum nanoparticles	AFB1	0.03	[46]
Lateral flow immunochromatographic assay	Sprayed coupled antigens AFB1-ovalbumin (AFB1-OVA) and ochratoxin A–ovalbumin (OTA-OVA)	AFB1	5.0	[47]
Noncompetitive immunocomplex immunoassay	Monoclonal capture antibody and a unique anti-immunocomplex antibody fragment isolated from a synthetic antibody repertoire	AFB1	0.07	[48]
Lateral flow immunochromatographic assay	mAbs-AuNP	AFB1	1.0	[49]
Dual immunochromatographic test strip	Double antibodies labeled with time-resolved fluorescent microspheres	AFM1	0.018	[37]
Gold immunochromatographic test strip	mAbs-AuNP	AFB1	0.5	[50]
Lateral flow immunochromatographic assay	Avi-tag (avidin tag)/streptavidin-oriented coupling strategy	AFB1	0.095	[51]

### 3.4. Electrochemical Biosensor-Based Methods

Electrochemical biosensors combine biorecognition elements (e.g., antibodies, aptamers) with electrochemical transducers to enable highly sensitive detection of aflatoxins [52,53]. These platforms include impedimetric, amperometric, and voltammetric sensors. Recent advancements have introduced polymer-based biosensors and portable devices integrated with smartphone technologies [54,55].

Despite their high sensitivity and rapid performance, biosensors often require customized fabrication and skilled operation, which currently limits their widespread adoption. Selected examples are presented in Table 5.

#### 3.4.1. Ultrasensitive Devices Based on Polymer-Based Biosensors

The future of electrochemical biosensor-based methods lies in the development of mobile, user-friendly, and cost-effective devices with enhanced sensitivity and lower detection limits for aflatoxins. High-sensitivity devices enable early detection of toxins, allowing timely interventions to reduce fungal contamination and eliminate associated metabolites.

An ultrasensitive plastic optical fiber (POF) biosensor coated with polyaniline has been developed for the detection of AFB1 [54]. Tested on a variety of matrices, including nuts, grains, beer, and biological fluids (e.g., serum and urine), the sensor demonstrated limits of detection (LOD) ranging from 0.061 µg/kg (peanuts) to 0.112 µg/kg (serum). The POF biosensor shows great promise due to its ease of use, replaceable cartridges, and high sensitivity.

Magnetic relaxation switching (MRS) immunosensors utilizing polystyrene beads and 150 nm superparamagnetic nanoparticles (SMRs) have also been introduced [55]. These sensors can detect AFB1 in wheat and corn, with a quantification range of 0.02–200 ng/mL and a detection limit of 14.3 pg/mL, representing exceptional sensitivity for trace-level detection.

Ultrasensitive polymer-based biosensor devices enable rapid and highly sensitive detection of aflatoxins, thereby enhancing food safety and minimizing economic losses [65]. Although these devices may be more expensive than simpler biosensors due to the use of advanced materials and technologies, they are more cost-effective in the long term compared to conventional analytical methods, such as chromatography or spectroscopy, owing to lower operational costs and minimal maintenance requirements.

#### 3.4.2. Sensitive Portable Devices

A key advancement in electrochemical biosensor-based methods is the development of sensitive portable devices designed for mobility, rapid measurement, and high analytical sensitivity. These devices are often more cost-effective than traditional laboratory-based instruments, such as chromatographs and spectrometers.

One promising approach for AFB1 detection involves the use of DNA-based intelligent hydrogels incorporating aptamers: single-stranded DNA or RNA molecules capable of binding specific targets with high affinity. Aptamers serve as versatile tools for detecting small molecules, including toxins, drugs, and environmental contaminants [66]. A DNA aptamer specifically targeting AFB1 has been developed, and a hydrogel system incorporating these aptamers along with gold nanoparticles (AuNPs) was constructed. Upon AFB1 binding, the hydrogel undergoes degradation, releasing AuNPs and inducing a visible color change in the solution. This method achieved a detection limit of 0.55 µg/kg and is well suited for portable, point-of-care testing (POCT) applications.

A smartphone-powered mobile microfluidic lab-on-fiber device (SMILE) employing immunoassay techniques has also been introduced for rapid, on-site quantification of AFB1 in feed samples [38]. This device integrates a nanobiosensor into an optical fiber and is powered by a smartphone, enabling a detection limit of 0.08 µg/L. The analysis is completed in just 12 min, offering high sensitivity, specificity, and reproducibility.

Furthermore, the immunochromatographic strip test has been improved through the incorporation of biotinylated nanobodies and a dual-probe signal amplification system [67]. This method achieved an ultralow detection limit of 0.03 ng/mL, providing fourfold greater sensitivity than traditional strip tests. It remains simple, rapid, and cost-effective, making it a promising tool for on-site AFB1 detection in diverse settings.

### 3.5. Chromatographic Methods

Chromatographic techniques, including high-performance liquid chromatography (HPLC), liquid chromatography–tandem mass spectrometry (LC-MS/MS), and gas chromatography–mass spectrometry (GC-MS), remain the gold standard for aflatoxin analysis due to their high precision and reliability [68,69,70]. Sample preparation typically involves extraction methods such as QuEChERS (quick, easy, cheap, effective, rugged, and safe) or solid-phase extraction.

Although chromatographic methods offer unparalleled sensitivity and analytical accuracy, they are labor-intensive, expensive, and require advanced laboratory infrastructure. Applications across various food matrices are summarized in Table 6.

### 3.6. Spectroscopic Methods

Spectroscopic techniques, including infrared (IR), near-infrared (NIR), fluorescence, and Raman spectroscopy, enable non-destructive and rapid screening of aflatoxins [83,84,85].

Often integrated into automated food sorting systems, these methods provide real-time analysis with minimal sample preparation, offering a practical alternative to traditional manual inspection. Applications of spectroscopic methods are summarized in Table 7.

### 3.7. Summary of Detection Methods

Recent advances in detection technologies have significantly improved aflatoxin monitoring. Emerging biosensor platforms, smartphone-integrated assays, and intelligent packaging represent promising future directions, aiming to provide faster, more affordable, and portable solutions for aflatoxin detection.

**Figure 2:**
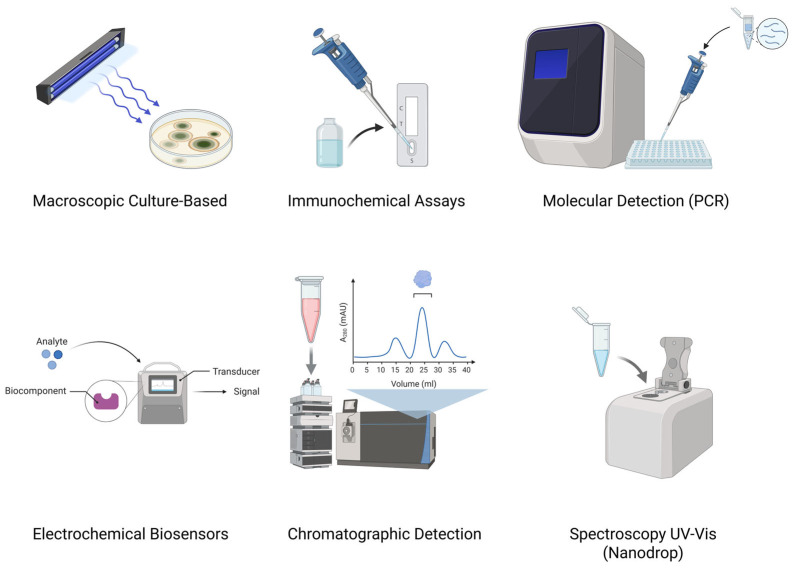
Schematic representation of major aflatoxin detection methods (top left to bottom right): macroscopic culture-based, molecular (PCR), immunochemical (lateral flow), electrochemical biosensors, chromatographic (HPLC, LC-MS/MS), and spectroscopic techniques (UV-vis Nanodrop).

These technological developments have enhanced food and feed safety surveillance by offering a wide array of tools with varying levels of sensitivity, specificity, cost, and applicability in field settings. Figure 2 presents a schematic overview of six core detection strategies commonly employed for identifying aflatoxins in diverse matrices.

The first approach, macroscopic culture-based methods, relies on the visual inspection of fungal colonies grown on selective media under ultraviolet light. Toxigenic *Aspergillus* strains are typically identified by their characteristic blue fluorescence. Although this technique is simple and inexpensive, it is time-consuming and primarily suited for preliminary screening.

In contrast, molecular techniques, such as conventional PCR and quantitative PCR (qPCR), target key genes involved in aflatoxin biosynthesis (e.g., *aflR*, *aflD*). These methods offer high sensitivity and enable early detection of potentially toxigenic fungi; however, they do not quantify actual toxin levels and require advanced laboratory infrastructure.

Immunochemical assays, including ELISA and lateral flow immunoassays, allow direct detection of aflatoxins via antigen–antibody interactions, typically generating a colorimetric or fluorescent signal. These tests are rapid, user-friendly, and cost-effective, making them suitable for on-site applications. Nevertheless, their semiquantitative nature and susceptibility to matrix interferences may affect analytical precision.

Electrochemical biosensors represent a rapidly evolving domain. These devices combine biorecognition elements (e.g., antibodies, aptamers) with electrochemical transducers, offering excellent sensitivity and potential for miniaturization. Despite their promise, they are not yet widely adopted due to fabrication complexity and cost.

Among traditional methodologies, chromatographic techniques, such as HPLC and LC-MS/MS, remain the gold standard for confirmatory analysis. These approaches offer precise quantification and reproducibility across multiple toxin types, but are limited by labor-intensive sample preparation, high costs, and the need for skilled personnel.

Lastly, spectroscopic methods, including infrared (IR), near-infrared (NIR), fluorescence, and Raman spectroscopy, enable rapid, non-destructive screening. Commonly integrated into automated sorting systems, these techniques are suitable for high-throughput analysis, although their sensitivity is limited when detecting trace concentrations of aflatoxins.

Collectively, these methods illustrate a dynamic and rapidly evolving field, with growing emphasis on portability, cost-effectiveness, and user accessibility, particularly in low-resource settings and high-risk regions.

### 3.8. Comparative Summary of Aflatoxin Detection Methods

A wide range of analytical methods has been developed for aflatoxin detection, each offering distinct advantages and limitations depending on the context of application. While certain techniques provide high sensitivity and specificity required for regulatory compliance, others are optimized for rapid, on-site screening or cost-effective monitoring in resource-constrained settings. To facilitate the selection of the most appropriate method, Table 8 provides a comparative overview of the main detection techniques currently employed in aflatoxin analysis. This comparison outlines key parameters such as sensitivity, complexity, speed, and suitability for field application.

Although numerous detection methods are available, each entails trade-offs among sensitivity, speed, cost, and field applicability. Immunochemical and biosensor-based approaches are increasingly favored for point-of-care applications, whereas chromatographic techniques remain the gold standard for regulatory confirmation. However, their practical implementation—especially in resource-limited settings—remains constrained. Future developments should focus on affordable, accurate, and portable devices that require minimal technical expertise.

## 4. Inhibitory Agents

**Figure 3:**
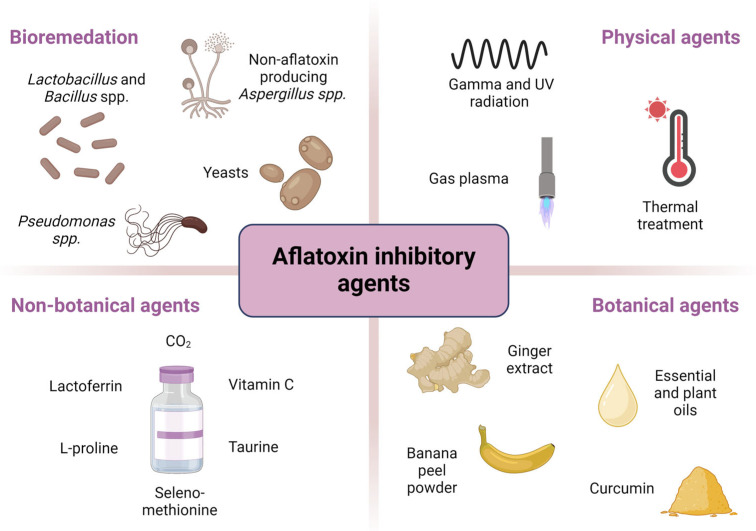
Overview of aflatoxin inhibitors and *Aspergillus* species: classification and examples of agents.

This paper proposes a classification system for aflatoxin inhibitors and *Aspergillus* species responsible for aflatoxin production, grouping them into four categories: bioremediation agents, physical agents, botanical agents, and non-botanical chemical agents. Figure 3 provides a visual summary of this classification along with representative examples for each category.

### 4.1. Bioremediation Agents

Bioremediation—the use of biological organisms to detoxify aflatoxins—offers a cost-effective and environmentally sustainable alternative to conventional decontamination methods [108]. This strategy is favored due to its ecological safety, economic viability, broad applicability to a wide range of mycotoxins, and its potential to produce minimal or no toxic by-products or intermediates [109,110,111].

Recent studies have identified various bacterial strains (e.g., *Bacillus*, *Pseudomonas, Lactobacillus*) capable of inhibiting *Aspergillus* growth and degrading AFB1 by up to 90% in vitro [9,111,112]. Some strains, such as *Lactobacillus rhamnosus* and *Kluyveromyces marxianus*, have demonstrated efficacy in animal models, significantly reducing aflatoxin absorption and toxicity [113,114].

However, the underlying degradation mechanisms remain poorly understood, highlighting the need for further investigation to enhance degradation efficiency and practical applicability in real-world settings [115]. A detailed overview of bioremediation agents—including their target mechanisms and reported efficacy—is provided in Table 9, summarizing strains shown to inhibit *Aspergillus* growth, suppress aflatoxin biosynthesis, or directly degrade aflatoxin molecules.

### 4.2. Physical Agents

Physical factors such as radiation, heat treatment, and atmospheric pressure plasma are employed to eliminate aflatoxins. Although heat treatment is commonly used, it demonstrates limited efficacy, as aflatoxins can withstand temperatures ranging from 237 °C to 306 °C [7]. For instance, boiling rice at 100 °C for 12 min reduced aflatoxin levels by 25%–56%, with brown rice showing the greatest reduction. However, the effectiveness of detoxification depends on several factors, including temperature, water volume, treatment duration, and initial contamination level.

Ultraviolet irradiation, known for degrading mycotoxins via photolysis and advanced oxidation processes, offers another potential strategy; however, aflatoxins are only minimally degraded by UVC (ultraviolet light, C band) radiation [6,128]. Gamma irradiation at doses up to 10 kGy (kilogray) has been shown to reduce AFB1 toxicity without significantly compromising food quality [129]. UVC has also demonstrated efficacy in reducing fungal growth and mycotoxin concentrations in rice [130], achieving over 70% reduction in AFB1 levels at doses of 4.88 J/cm^2^ [6].

Electron beam irradiation has similarly been applied to reduce aflatoxin levels in pistachios. Doses ranging from 4 to 6 kGy significantly decreased both *A. flavus* spore viability and AFB1 content, though higher doses may adversely impact product quality [19].

A novel and highly promising method is non-equilibrium cold atmospheric plasma (CAP), which produces reactive oxygen and nitrogen species capable of degrading complex chemical compounds [131]. CAP has demonstrated remarkable effectiveness, achieving a 96% reduction in AFB1 within 60 s, with no detectable residues after 120 s. This positions CAP as a highly viable technology for mycotoxin detoxification.

### 4.3. Non-Botanical Agents

Research indicates that the effects of mycotoxins may be mitigated through their interaction with non-botanical compounds. These substances can form non-toxic complexes with aflatoxins, such as the AFB2a–arginine adduct (AFB2a-Arg). However, it is essential to thoroughly evaluate the resulting transformation products, as they may retain genotoxic properties, exhibit new toxic effects, or potentially revert to aflatoxins under certain conditions [132]. Table 10 presents findings from studies involving non-botanical compounds.

Only a limited number of reports have investigated synthetic chemical compounds, likely due to the growing emphasis on “green chemistry” and the use of environmentally benign alternatives for aflatoxin elimination [133]. Although vitamin C is derived from plants, it is categorized here as a non-botanical agent due to its use in purified, isolated form, rather than as part of a whole plant extract.

**Table 10:** Non-botanical agents with antifungal and antitoxic effects on *Aspergillus* fungi and aflatoxins.

Aflatoxin and/or Fungus	Type of Non-Botanical Agent	Model	Effects	References
AFB1	Mixture of citric and phosphoric acids with arginine	in vitro	99% elimination of AFB1 from solution and corn kernels aflatoxin transformed into the AFB2a-Arg adduct	[132]
AFB1 AFM1	Lactoferrin	Caco-2, HEK 293, Hep-G2, and SK-N-SH cells in vitro	restoration of cell viability reduction in glutathione (GSH) and malondialdehyde (MDA) levels LDH release (dose-dependent) reduction in DNA damage to normal levels (at 1000 mg/mL) decreased ERK1/2 levels in Caco-2 and Hep-G2 cells decreased JNK expression in Hep-G2 and SK-N-SH cells	[134]
*A. parasiticus* ATCC15517 AFB1 AFB2 AFG1 AFG2	Vitamin C	in vitro	mycelium disturbances at 50 mg/mL *aflR* gene expression reduced to 68.7% and 81% at 25 and 50 mg/mL of vitamin C, respectively	[135]
*A. flavus* A42 and CHAO50	CO_2_	in vitro	high inhibition of mycelium growth at 100% CO_2_ in vacuum lowest levels of AFB1, AFB2, AFG1, AFG2, and total aflatoxins from A42 isolate at 100% CO_2_ lowest levels of AFB1, AFB2, AFG1, AFG2, and total aflatoxins in CHAO50 isolate at 100% CO_2_	[136]
AFB1 AFM1	L-proline	HEK 293 cells in vitro; mice in vivo	significant alleviation of abnormal biochemical parameters, renal pathology, and excessive cell apoptosis increased cell viability reduction in total antioxidant capacity (TAC) significant increase in MDA levels	[137]
AFB1	Chlorine dioxide gas	in vitro	post-treatment with ClO_2_: over 99.5% AFB1 degraded within 12 h low toxicity of degradation products	[138]
AFB1	Complementary feed *Rhino-Hepato Forte*	Chicken in vivo	after both doses, parameters like red blood cell count, packed cell volume, hemoglobin, white blood cell count, H/L stress ratio, triglycerides, cholesterol, alkaline phosphatase, creatinine, glucose, calcium, and total protein returned to normal	[139]
AFB1	Taurine	Rats in vivo	inhibition of liver damage markers and MDA increase increased hepatic TAC, SOD, GPx, CAT activity, and mitochondrial membrane potential reduction in liver pathological changes regulation of mRNA expression of Nrf2, NQO1, HO-1, and GCLC inhibition of Bcl-2 and Bcl-2/Bax expression decline increase in Bax, cleaved caspase 9, and cleaved caspase 3 expression	[140]
AFB1	Seleno-methionine	Rabbits in vivo	reduction in kidney damage more pronounced effect at 0.2 mg/kg than 0.4 mg/kg significant reductions in blood urea nitrogen, creatinine, uric acid, urinary protein, renal glycogen, and apoptotic cells significant increases in ROS, MDA, Keap1, and expression of Bax, caspase 3, caspase 9, and PTEN increased activity of antioxidant enzymes TAC, GPx, SOD, Nrf2, NQO1, HO-1, and expression of PI3K, Bcl-2, AKT, and p-AKT improvement in cell and glomerular structure, reduction in vacuoles	[141]

### 4.4. Botanical Agents

Synthetic fungicides have long been used to treat and prevent fungal infections in plants and stored seeds. However, growing consumer awareness of their potential negative effects on the environment and on human and animal health has led to increased interest in alternative plant protection methods [6]. Natural compounds and phytochemicals contain a variety of biologically active substances (e.g., phenols, steroids, glycosides, and alkaloids) that may help restore physiological homeostasis after ingestion of mycotoxin-contaminated products [142,143]. For instance, natural plant phenols can reduce oxidative damage without inducing side effects [144,145]. Moreover, certain raw plant materials have been shown to inhibit fungal growth [146], while others—such as banana peel powder [147] and jatropha pomace extract [148]—have demonstrated the ability to reduce aflatoxin levels.

Table 11 lists botanical agents with antitoxic and antifungal properties effective against aflatoxins and *Aspergillus* species, respectively. It is likely that many other plants and plant-derived products with similar potential remain unexplored and warrant further investigation.

### 4.5. Integrated Decontamination Strategies

A variety of physical, chemical, and biological strategies have been employed to reduce aflatoxin contamination in food and feed products. Each method presents specific advantages and limitations depending on the matrix, contamination level, and desired product quality.

Mechanical techniques such as sorting, cleaning, and optical separation have achieved aflatoxin reductions of up to 95%, although some product loss may occur during processing [168]. Thermal treatments are constrained by the high thermal stability of aflatoxins. Nonetheless, specific applications—such as cooking or steaming soybeans—have achieved reductions ranging from 33% to 97% [169]. Combinatorial approaches, such as heat treatment in the presence of citric acid, have shown enhanced efficacy, with one study reporting a 49.2% reduction in pistachios [170].

Irradiation methods, including UVC and electron beam exposure, have demonstrated promising results in degrading aflatoxins without compromising product quality. For instance, rotating peanuts during irradiation enhanced AFB1 degradation by 25% [171]. Similarly, cold plasma treatment—a non-thermal technique—achieved up to 96% AFB1 reduction within 60 s while preserving nutritional value [131].

Chemical agents such as citric, lactic, and tartaric acids have been shown to reduce aflatoxin levels by over 95% in certain nuts. Additionally, ozonation has achieved reductions in AFB1 concentration of up to 86.75% [132,172]. Biological materials—including yeast cell walls and specific bacterial strains—have demonstrated potential for aflatoxin binding, although further in vivo studies are needed to confirm their safety and efficacy in livestock [173,174]. Feed additives such as mineral clays and activated carbon are also commonly used to adsorb or neutralize mycotoxins in the gastrointestinal tract, thereby minimizing systemic absorption [175].

Despite promising outcomes, no single strategy provides a comprehensive solution. Considerations such as scalability, cost-effectiveness, regulatory approval, and impact on nutritional quality must guide implementation. Future directions may involve synergistic applications and biotechnological innovations that integrate efficiency, safety, and sustainability.

## 5. Occurrence of Aflatoxins in Water: Detection and Elimination Strategies

Water contamination—whether anthropogenic or natural—is a significant global concern, particularly in developing countries where poor sanitation and reliance on rivers, streams, wells, and lakes for drinking water are common. While most research has focused on chemical contaminants such as heavy metals and organic pollutants, mycotoxins in water—especially aflatoxins produced by fungi such as *Aspergillus* spp.—remain understudied. This issue is relevant in both developing and developed countries [176].

### 5.1. Detection of Aflatoxins in Water

Detection of aflatoxins in drinking and surface waters is essential. LC-MS/MS techniques have been employed to detect aflatoxins in bottled water, achieving detection limits as low as 0.2 ng/L [177]. Aflatoxins AFB2, AFB1, and AFG1 were found at trace levels that did not pose a health risk to adults. UHPLC-ESI-QTOF (ultrahigh-performance liquid chromatography–electrospray ionization–quadrupole time-of-flight mass spectrometry) was used to detect aflatoxins B1 and B2 produced by *Aspergillus fumigatus* in untreated water sources in Nigeria [178]. These concentrations, although initially detectable, diminished over time.

A multistage solid-phase extraction method coupled with HPLC-HRMS (high-performance liquid chromatography–high-resolution mass spectrometry) enabled the identification of aflatoxins in surface water from the Ter River, where AFB1 was detected only in trace amounts [179]. Innovative biosensors—such as Zr-LMOF@Cotton (zirconium-based luminescent metal–organic framework on cotton)—have demonstrated low detection limits (0.1 µg/L), rapid results, and cost-effectiveness [180]. A portable biosensor based on a DNA pyramid structure achieved detection limits as low as 3 pg/mL, comparable to more expensive HPLC-MS/MS systems [66].

Although food remains the primary route of aflatoxin exposure, water contamination should not be underestimated—especially in regions where untreated surface water is consumed. The growing detection of aflatoxins in aquatic environments highlights the need to expand monitoring programs. However, the ecological and toxicological consequences of chronic, low-level exposure via water are still poorly understood and require further research.

### 5.2. Elimination of Aflatoxins from Water

Given the increasing global threat posed by persistent organic pollutants, addressing aflatoxin contamination in water is essential. Considerable progress has been made in developing effective adsorbents and degradation techniques. UVA LED systems have been used to degrade aflatoxins B1 and M1 in water, achieving reductions of 70% and 84%, respectively, at a dose of 1200 mJ/cm^2^. Importantly, the degradation by-products were found to be non-toxic [181]. Zr-LMOF@Cotton material also demonstrated 92% removal efficiency and maintained over 95% efficiency across 10 reuse cycles [180]. β-Lactoglobulin-based aerogels have shown strong adsorption capacity and the ability to be regenerated for repeated use [182]. Additionally, modified *Spirogyra* biomass was effective in adsorbing aflatoxins, achieving a 91% reduction under optimized conditions [183].

### 5.3. Regulatory and Research Considerations

Despite well-established regulations for aflatoxins in food, no formal standards currently exist for water. Although aquatic concentrations are typically low, chronic exposure may lead to long-term environmental persistence and bioaccumulation. It is therefore necessary to evaluate both cumulative and long-term impacts on ecosystems and human health [178].

## 6. Perspectives on Detection and Elimination of Aflatoxins

Preventing mycotoxin contamination—particularly by aflatoxins—is a key priority in food safety initiatives due to their well-documented adverse health effects [175]. Climate change is exacerbating the risk of aflatoxin contamination, underscoring the need for effective mitigation strategies across all stages of the food supply chain, from crop cultivation and harvesting to food processing, packaging, and storage.

The One Health approach, which integrates human, animal, and environmental health, promotes the development of comprehensive strategies to manage such risks [184]. This framework includes improving agricultural practices, strengthening food safety protocols, and addressing environmental conditions conducive to fungal proliferation, such as rising temperatures and increased humidity [185].

In Latin America, although the One Health concept is still being formalized, initiatives based on its principles have been implemented for over a decade—particularly in rural and underserved urban areas—yielding success in disease prevention and control even prior to its formal adoption [186]. In contrast, countries like India face implementation challenges, primarily due to insufficient interministerial coordination [187]. Conversely, Rwanda has made notable progress by introducing national policies, establishing surveillance laboratory networks, and mobilizing community health workers to support integrated efforts under the One Health framework [186].

**Figure 4:**
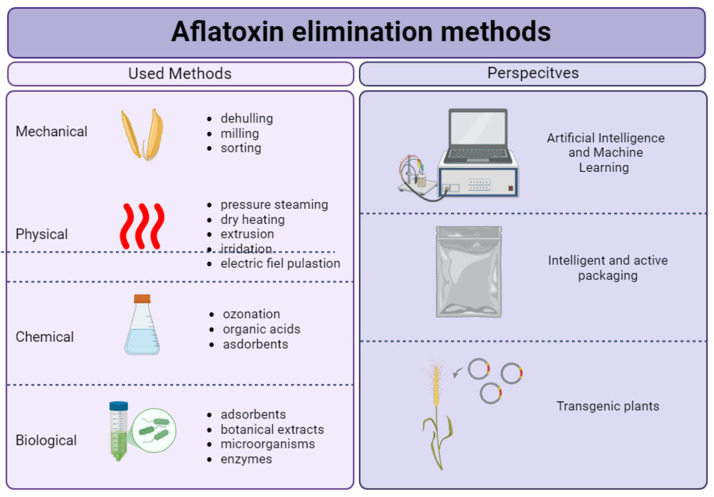
Methods of aflatoxin elimination: current practices and future trends.

Given these developments, continued innovation in aflatoxin elimination methods is essential. Three future-oriented approaches are illustrated in Figure 4.

### 6.1. Artificial Intelligence (AI) and Machine Learning (ML)

Artificial intelligence (AI) and machine learning (ML) are rapidly advancing and becoming essential tools in the fight against food contamination. AI enables the analysis of large datasets, facilitates predictive modeling, and supports process optimization—capabilities that were previously unattainable. For example, AI can be used to monitor environmental conditions in storage facilities to predict and mitigate fungal contamination. The integration of sensors with the Internet of Things (IoT) and machine learning algorithms has demonstrated the ability to monitor grain quality and prevent fungal growth [108]. By analyzing parameters such as temperature, humidity, and CO_2_ concentration, these systems help maintain optimal storage conditions and significantly reduce contamination risk over extended periods.

AI technologies also support food safety management by enhancing supply chain efficiency and reducing the risk of disease transmission [188]. Moreover, AI contributes to aflatoxin detection. For instance, Raman spectroscopy combined with machine learning algorithms has demonstrated high accuracy in detecting aflatoxins in maize [189]. AI-driven computational tools are also aiding in the identification of microbial strains and enzymes capable of detoxifying mycotoxins, thereby advancing food safety research [190]. These developments underscore the potential of AI and ML to streamline detection protocols, reduce response time, and optimize safety interventions.

### 6.2. Intelligent and Active Packaging (AP)

Packaging technology plays a vital role in ensuring food safety, particularly in preventing contamination by mycotoxins such as aflatoxins. Hermetic packaging, such as Purdue improved crop storage bags, has been shown to effectively reduce aflatoxin levels in maize [191]. These solutions represent a cost-effective and eco-friendly alternative to chemical preservatives by creating unfavorable conditions for fungal growth and toxin production [192].

Intelligent and active packaging has emerged as a promising area of innovation. Intelligent packaging incorporates sensors that monitor parameters such as temperature and humidity, providing real-time feedback on product quality. Active packaging, in contrast, integrates antimicrobial agents, antioxidants, or essential oils to actively inhibit microbial growth and extend shelf life [193]. Studies have demonstrated the effectiveness of active antifungal packaging in controlling fungal contamination. For example, natural extracts such as bee bread [194] and essential oils derived from cinnamon, anise, and orange [195] have exhibited antifungal properties when incorporated into packaging films.

Active packaging has also shown efficacy in inhibiting *Aspergillus* spp., the primary producers of aflatoxins. In bread, coatings with ε-poly-L-lysine have been shown to suppress *Aspergillus parasiticus* [196], while chitosan coatings applied to peanuts effectively reduced fungal proliferation and aflatoxin production [197]. Similarly, probiotic coatings based on sodium alginate have successfully inhibited *Aspergillus* growth in soft cheeses [198]. These innovative approaches not only reduce fungal contamination but also improve shelf life and reduce food waste, thereby supporting sustainability.

### 6.3. Transgenic Plants

Although the complete elimination of aflatoxins remains challenging, transgenic plants have emerged as a promising strategy to mitigate contamination. First and second generations of genetically modified (GM) crops were developed to enhance resistance to fungal infection and reduce aflatoxin production [199]. For instance, the introduction of the synthetic peptide AGM182 into maize has resulted in over 70% reduction in aflatoxin contamination [200]. This peptide, modeled after tachyplesin 1 from the Japanese horseshoe crab, demonstrated fivefold greater efficacy against *Aspergillus flavus* than its natural analogue. Despite these advances, transgenic maize still often contains aflatoxin levels above regulatory limits, underscoring the need for further genetic refinement.

Another innovative approach is host-induced gene silencing (HIGS), which targets and suppresses fungal genes responsible for aflatoxin biosynthesis. For example, maize engineered with RNA interference (RNAi) targeting *aflC* exhibited undetectable aflatoxin levels following *A. flavus* infection [201]. This method has proven effective without compromising plant development. Additional studies have focused on silencing the *polygalacturonase* gene of *Aspergillus flavus*, with transgenic maize showing significantly reduced aflatoxin accumulation [202].

While genetically modified crops offer considerable potential for reducing aflatoxin contamination, global adoption remains limited due to regulatory restrictions and public skepticism [199]. Continued research into novel genetic strategies and refinement of existing technologies are essential for enhancing food safety and crop resilience.

Technological advancements are reshaping aflatoxin control; however, many of these innovations face challenges related to scalability and practical implementation. Although artificial intelligence, intelligent packaging, and transgenic crops demonstrate significant potential, their widespread adoption is constrained by economic, regulatory, and societal barriers. Integration of such technologies within a One Health framework may facilitate more holistic and sustainable solutions. Future progress will depend on interdisciplinary collaboration and the development of user-centered, adaptable systems.

## 7. Future Perspectives

Despite significant progress in the detection and mitigation of AFB1, several challenges remain unresolved. Climate change—characterized by rising temperatures and increased humidity—is expected to expand the geographical range of aflatoxin contamination, impacting crops in regions previously considered low-risk. As a result, future strategies must prioritize the development of robust, climate-resilient interventions.

Detection technologies should continue evolving towards miniaturization, automation, and field-deployable applications. The integration of biosensors with artificial intelligence and smartphone-based systems holds promise for creating highly sensitive, portable, and cost-effective detection tools, particularly suitable for low-resource settings. Moreover, intelligent packaging materials equipped with real-time aflatoxin sensors may revolutionize food safety monitoring throughout supply chains.

On the mitigation side, increasing attention should be directed toward the discovery of novel bioremediation agents capable of degrading aflatoxins effectively under diverse environmental conditions. Advances in genomics and synthetic biology may enable the design of microbial strains or enzymatic systems specifically tailored for aflatoxin detoxification. Transgenic crop development—especially utilizing CRISPR-Cas9 (clustered regularly interspaced short palindromic repeats–CRISPR-associated protein 9)—also shows promising potential for enhancing crop resistance to aflatoxin-producing fungi. However, despite their scientific promise, these approaches remain largely experimental and face considerable limitations, including regulatory hurdles, biosafety concerns, public skepticism, and implementation costs. At present, their application is largely confined to research environments, and further translational studies are needed to enable real-world deployment. Therefore, such innovations should be regarded as complementary rather than substitutes for existing mitigation strategies.

A more holistic, interdisciplinary approach that integrates improved agricultural practices, technological innovation, regulatory enforcement, and public awareness initiatives is essential. Strengthening international collaboration and harmonizing global aflatoxin standards will be pivotal in enhancing food safety and protecting public health amid accelerating environmental and socioeconomic change.

The economic feasibility of aflatoxin detection and mitigation strategies must also be considered. While advanced technologies such as LC-MS/MS and AI-enhanced biosensors offer exceptional sensitivity, their costs may limit adoption in resource-constrained regions. Thus, future efforts should aim to balance analytical performance with affordability. Cost–benefit analyses will be crucial to guide the transition from laboratory innovation to field and industrial applications.

Effective aflatoxin control inherently requires interdisciplinary cooperation. Successful initiatives often involve collaboration among microbiologists, engineers, data scientists, policymakers, and agricultural stakeholders. For instance, implementing IoT-based monitoring systems in storage facilities has required expertise in both agronomy and information technology. Similarly, progress in transgenic crop development has relied on molecular biologists working alongside regulatory and food policy experts. Strengthening such cross-disciplinary partnerships will be vital for developing scalable, sustainable, and equitable aflatoxin management strategies.

Looking ahead, successful aflatoxin control will hinge on the convergence of technological innovation and practical implementation. Emerging solutions must be evaluated not only for analytical accuracy but also for cost-effectiveness and long-term sustainability. Empowering local stakeholders—from farmers to food processors—and fostering cross-sectoral collaboration will be critical. The future of aflatoxin management lies in adaptive, context-specific, and globally coordinated action.

## 8. Conclusions

Aflatoxin B1 remains one of the most significant threats to global food and feed safety, with its impact intensified by climate change and the complexities of international trade. Over the last decade, notable progress has been achieved in the detection, mitigation, and inhibition of AFB1, ranging from conventional analytical techniques to advanced biosensor and AI-assisted systems. Strategies such as bioremediation, physical and chemical decontamination, and botanical interventions have shown promise in reducing aflatoxin contamination levels. Nevertheless, each method presents certain limitations in terms of cost, scalability, sensitivity, and practical applicability.

An integrated, multifaceted approach—combining technological innovations in detection with sustainable and accessible mitigation measures—is essential to effectively manage aflatoxin risks across diverse environmental and socioeconomic contexts. Ongoing interdisciplinary collaboration among microbiologists, chemists, engineers, and data scientists will be critical in overcoming the persistent challenges posed by AFB1 contamination and in ensuring long-term food safety.

## Figures and Tables

**Table 1:** Standards for aflatoxins in animal feed established by Missouri Department of Agriculture [22].

AF Level [μg/kg]	Class of Animal	Commodities
20	Dairy animals, animals not specified in other categories, or animals with unknown use	For corn, peanut products, cottonseed meal, and other animal feeds and feed ingredients
20	Immature animals	For corn, peanut products, and other animal feeds and feed ingredients, excluding cottonseed meal
20	Pets of all ages (e.g., dogs, cats, rabbits)	For corn, peanut products, cottonseed meal, other food ingredients, and complete pet food
100	Breeding cattle, breeding swine, and mature poultry (e.g., laying hens)	For corn and peanut products
200	Finishing swine (weighing 100 pounds or more)	For corn and peanut products
300	Beef cattle, swine, and poultry (regardless of age or breeding status)	For cotton seed meal
300	Finishing beef cattle (e.g., feedlot cattle)	For corn and peanut products

**Table 2:** Standards for AFB1 in animal feed established by the Chinese government [22].

Feedstuff		AB1 Level [μg/kg]
**Raw** **materials**	Maize processing products, peanut meal	≤50
	Vegetable oil	≤10
	Maize oil, peanut oil	≤20
	Other plant-based feed materials	≤30
**Products**	Concentrated feed for piglets and young birds	≤10
	Concentrated feed for meat ducks, growing ducks, and ducks for egg production	≤15
	Other concentrated feed	≤20
	Calf and lamb concentrate supplement	≤20
	Concentrate supplement for lactation	≤10
	Other concentrate supplements	≤30
	Compound feed for piglets and young birds	≤10
	Compound feed for meat ducks, growing ducks, and laying ducks	≤15
	Other formula feed	≤20

**Table 3:** Molecular methods used to identify contaminated food.

Method	Amplified Gene	Detected Strain	Limit of Detection	References
Polymerase chain reaction	*aflP*	Aflatoxigenic strain	Not calculated	[31]
Real-time polymerase chain reaction	*aflD*, *aflM* (*ver*), *aflP*, *aflQ*	Aflatoxigenic strain	Not calculated	[33]
Polymerase chain reaction and real-time polymerase chain reaction	*aflR*, *aflM* (*ver*), *aflD* (*nor*)	Aflatoxigenic strain	Not calculated	[34]
Polymerase chain reaction	*afM* (*ver*), *aflJ*, *afIR*, *afID* (*nor*)	Aflatoxigenic strain	Not calculated	[30]
Loop-mediated isothermal amplification and real-time polymerase chain reaction	*aflT*	Aflatoxigenic strain	100-999 picograms of DNA (loop-mediated isothermal amplification) 160 femtograms of DNA (qPCR)	[32]

**Table 5:** Electrochemical biosensors used to detect aflatoxins in food.

Method	Detection Matrix	Detection Method	Detected Aflatoxin	Limit of Detection for AFs [μg/kg]	References
Fluorescent aptamer	Fluorescently labeled aptamers bind aflatoxins, blocking DNA nanostructures and quenching fluorescence	Fluorescence	AFB1, AFM1	0.009 0.00624	[56]
MiSens biosensor chip	MiSens silicone dioxide biochip consisting of 2 sets of Au electrode arrays with 3 working electrodes each, surface modified with protein A–aflatoxin antibody conjugate	MiSens and HPLC (high-performance liquid chromatography)	AFB1	390	[57]
Electrochemical immunosensor using modified MWCNTs/CS/SPCE	Multi-walled carbon nanotubes/chitosan/screen-printed carbon electrode	Differential pulse voltammetry	AFB1	0.0003	[58]
Flexible, dispense-printed electrochemical biosensors	Dispense-printed electrodes, which are functionalized with single-walled carbon nanotubes and subsequently coated with specific antibodies	Chronoamperometric	AFM1	0.02	[59]
Anti-idiotypic nanobody proximity-dependent immuno-polymerase chain reaction (PD-IPCR)	Two phages displaying the variable domain of the heavy chain anti-idiotypic nanobody that binds to an aflatoxin- or zearalenone-specific monoclonal antibody	Real-time quantitative polymerase chain reaction	Total AFs	0.03	[60]
Luminescence method using ATP-releasing nucleotides	Magnetic bead aptamer complex	Luminescence	AFB1	0.000009	[61]
Hyperbranched gold plasmonic blackbody- enhanced immunochromatographic test strip	Hyperbranched gold plasmonic blackbody	Optical density of test and control line	AFB1	0.016	[62]
Self-replicating catalyzed hairpin assembly	Hairpin auxiliary probes, H1 and H2	Fluorescence	AFB1	0.13	[63]
Gd-MOF/USPIO magnetic field sensor	Gadolinium-based metal–organic framework (Gd-MOF) and ultrasmall superparamagnetic iron oxide (USPIO)	Magnetic resonance	AFB1	0.00054	[64]

**Table 6:** Chromatographic methods used to detect aflatoxins in food.

Method	Detector	Detected Aflatoxins	Limit of Detection for AFs [μg/kg]	References
High-performance liquid chromatography	Q-Trap 5500 LC-MS/MS system with a turbo ion spray source	AFB1, AFB2, AFG1	0.04–0.05	[68]
Liquid chromatography	TSQ Quantum Discovery system: a high-performance triple-stage quadrupole mass spectrometer with electrospray ionization	AFB1, AFG1, AFG2	0.04	[69]
Liquid chromatography–tandem mass spectrometry	Triple-quadrupole mass spectrometer with an electrospray ionization source	AFB1	0.03	[71]
High-performance liquid chromatography	Fluorescence detector	AFM1	0.002	[72]
Online solid phase extraction coupled with ultra-high performance liquid chromatography	Triple-quadrupole mass spectrometer	AFM1	0.0007	[73]
Ultrahigh-performance liquid chromatography	Triple-quadrupole electrospray ionization mass spectrometer	AFB1, AFB2, AFG1, AFG2	0.25–0.32	[74]
Ultrahigh-performance liquid chromatography	Quadrupole orbitrap mass spectrometer	AFB1, AFB2, AFG1, AFG2, AFM1	0.0003–0.0080	[75]
Liquid chromatography	Triple-quadrupole mass spectrometer with an electrospray ionization source	AFB1, AFB2, AFG1, AFG2, AFM1	0.02–10.14	[76]
Ultrahigh-performance liquid chromatography	Tandem-quadrupole mass spectrometer	AFB1, AFB2, AFG1, AFG2, AFM1, AFM2	0.14	[77]
Ultrahigh-performance liquid chromatography	Fluorescence detector	AFB1, AFB2, AFG1, AFG2	0.07 0.08 0.06 0.09	[78]
High-performance liquid chromatography	Fluorescence detector	AFB1, AFB2, AFG1, AFG2,	0.1	[79]
High-performance liquid chromatography	Fluorescence detector	AFB1	16.5	[80]
Quantitative thin-layer chromatography	Thin layer chromatography scanner (fluorescence)	AFB1, AFB2, AFG1, AFG2	<2.0	[81]
Ultrahigh-performance liquid chromatography	Fluorescence detector	AFB1, AFB2, AFG1, AFG2	2	[82]

**Table 7:** Spectroscopic methods used to detect aflatoxins in food.

Method	Detected Aflatoxin	LOD for AFs [μg/kg]	Ref.
Fluorescence spectroscopy and multispectral imaging	AFB1	Not calculated	[86]
Laser-induced fluorescence spectroscopy	Aflatoxins	Not calculated	[87]
Fourier transform near-infrared reflectance spectroscopy	AFB1, AFB2, AFG1, AFG2	<4.0	[88]
Colorimetric competitive enzyme immunoassay	AFB1	0.1	[89]
Attenuated total reflectance–Fourier transform infrared (ATR-FTIR) spectrometry	AFM1	0.02	[90]
Custom-built ultraviolet-visible–near infrared spectroscopy system (UV-vis–NIR)	Aflatoxins	Not calculated	[91]
Near-infrared and mid-infrared spectroscopy with chemometrics	AFB1, AFB2, AFG1, AFG2	Not calculated	[92]
Multiplexing fiber optic laser-induced fluorescence spectroscopy system with one-, two-, and three-probe	AFB1	Not calculated	[93]
Fluorescence spectroscopy	AFB1, AFB2	0.2	[94]
Shortwave near-infrared spectroscopy	AFB1	Not calculated	[95]
Visible-near-infrared spectroscopy	AFB1, aflatoxins	Not calculated	[96]
Terahertz spectroscopy with chemometric methods	AFB1	1.0	[97]
On-line fluorescence spectroscopy system	AFB1	<6.20	[98]
Hyperspectral imaging technology (UV)	Aflatoxins	Not calculated	[99]
Near-infrared reflectance spectroscopy-based fast versicolorin A detection	AFB1	8.26	[100]
Laser-induced fluorescence spectroscopy	AFB1	6.86	[101]
Fourier transform infrared spectroscopy	AFM1	10.0	[102]
Colorimetric enzyme-linked immunosorbent assay	AFB1	0.06	[103]
Ultraviolet-visible–near-infrared spectroscopy	Aflatoxins	Not calculated	[104]
Fluorescence spectroscopy and multispectral imaging	AFB1	Not calculated	[105]
Fourier transform near-infrared spectroscopy	AFB1	Not calculated	[106]
Handheld fluorescence spectrometry	AFB1	0.6	[107]

**Table 8:** Comparative overview of aflatoxin detection methods: principles, advantages, and limitations.

Detection Method	Advantages	Limitations
**Macroscopic Culture-Based**	Low cost; simple setup; suitable for preliminary fungal screening	Time-consuming (32–120 h); requires microbiological expertise; not specific to toxins
**Molecular Techniques**	High sensitivity and specificity; enables detection of toxigenic strains	Does not detect actual toxin levels; costly equipment; technical expertise required
**Immunochemical**	Rapid; low cost; suitable for screening; can detect multiple toxins simultaneously	Cross-reactivity; matrix effects; lower quantitative accuracy; may need confirmation
**Electrochemical Biosensors**	High sensitivity; miniaturizable; potential for on-site use	Limited standardization; device-specific calibration; still in early-stage commercialization
**Chromatographic**	Gold standard; precise quantification; multi-analyte detection	Expensive instrumentation; time-consuming sample preparation; requires trained personnel
**Spectroscopic Techniques**	Rapid; non-destructive; no need for chemical reagents	Lower sensitivity; affected by matrix complexity; limited detection of low toxin concentrations

**Table 9:** Bioremediation agents blocking the growth of *Aspergillus*, inhibiting aflatoxin secretion, or causing aflatoxin degradation.

Aflatoxin and/or Fungus	Inhibitor	Model	Effects	References
*A. parasiticus*; aflatoxins	*Lactobacillus plantarum* *L. delbrueckii subsp. Lactis*	in vitro	growth inhibition of *A. parasiticus* by both strains aflatoxin production reduction inhibition of *aflR* expression	[116]
AFB1 AFB2 AFM1	*Bacillus shackletonii* strain L7	in vitro	aflatoxin level reduction: AFB1 by 92.1%, AFB2 by 84.1%, AFM1 by 90.4% L7 culture supernatant more effective than live cells or extracts	[112]
AFB1	*Pseudomonas fluorescens* strain 3JW1	in vitro	significant inhibition of *A. flavus* growth and aflatoxin production 88.3% degradation of AFB1	[9]
*A. parasiticus* AFB1 AFG1	*Bacillus* *amyloliquefaciens* UTB2 *B. subtilis* UTB3	in vitro	inhibition of *A. parasiticus* growth inhibition of AFB1 and AFG1 production disruption of mitochondrial and cell membrane functions alterations in the shape and structure of fungal hyphae and vesicles	[117]
AFB1	*Bacillus velezensis*	in vitro	AFB1 degradation of 91.5% highest degradation capacity of the supernatant	[111]
*A. flavus* *A. niger* AFB1	*Lactobacillus* *plantarum* strain ITEM 17215	in vitro	growth inhibition of both fungal species reduced aflatoxin B1 bioavailability	[118]
*A. parasiticus* AFB1	*Bacillus* *mojavensis* RC1A *B. subtilis* RC6A	in vitro	slowed *A. parasiticus* growth reduced AFB1 production *B. mojavensis* RC1A strain exhibits the highest inhibition of fungal growth	[119]
*A. flavus* AFB1	Volatile organic compounds from *Streptomyces alboflavus* TD—1	in vitro	complete inhibition of *A. flavus mycelium*, embryos, and conidial germination inhibition AFB1 secretion	[120]
AFB1	*Lactobacillus* *rhamnosus* GG	Dairy cattle in vivo	mitigated growth-retardant effect in calves reduced activity of aspartate aminotransferase (AST) and lactate dehydrogenase (LDH) reduced AFB1 absorption in the gastrointestinal tract increased excretion of AFB1 and AFM1 in feces and urine significant reduction in AFB1 and AFM1 toxicokinetics in calf urine	[113]
AFB1 AFB2 AFG1 AFG2	Recombinant laccase C30 from *Saccharomyces cerevisiae*	in vitro	binding affinity to aflatoxins: AFB1 > AFG2 > AFG1 > AFB2 increased AF degradation with prolonged incubation time	[121]
*A. flavus* *A. parasiticus* AFB1 AFB2 AFG1 AFG2	*Lactobacillus* *rhamnosus* *L. gasseri* *L. plantarum*	in vitro	On YES media: maximum reduction of aflatoxins produced by *A. parasiticus* with *L. rhamnosus* strain reduced reduction rate of AFs produced by *A. flavus* On wheat grains: optimal inhibition of aflatoxin secretion produced by *A. parasiticus* with *L. rhamnosus* strain	[122]
AFB1 AFM1	*Kluyveromyces marxianus* CPY1 *K. marxianus* RSY5 *Pichia kudriavzevii* YSY2	Dairy cattle in vivo	72.08% reduction in AFM1 concentration lowest AFM1 levels following CPY1 and RSY5 strain administration enhanced dry matter intake and milk composition decreased transfer of AFB1 from feed to AFM1 in milk	[114]
AFM1	*Lactobacillus* *paracasei* 108 *L. plantarum* 49 *L. fermentum* 111	in vitro	reduction by live isolates (73.9%–80.0%) and nonviable isolates (72.9%–78.7%) *L. paracasei* 108 strain exhibits the highest AFM1 removal capacity in both living and non-living cells	[123]
AFB1	*Lactobacillus* *plantarum*	in vitro	90% AFB1 reduction during fermentation	[124]
AFB1 AFB2 AFG1 AFG2	*Aspergillus oryzae* *A. versicolor* *A. ochraceus* *Cladosporium subcinereum* *Bacillus* *albus* *B. velezensis*	Human skin fibroblast cells in vitro	aflatoxin reductions after one year of fermentation: 92.58% (AFB1), 100% (AFG1), 98.69% (AFB2), 100% (AFG2)	[125]
*A. flavus* AFB1	*Bacillus* *amyloliquefaciens* WF2020	in vitro	minimal inhibition of fungal growth significant or complete AFB1 degradation, dependent on concentration reduced expression of 10 aflatoxin pathway genes and two transcription factors (*aflR, aflS*) in *A. flavus*	[126]
*Aspergillus flavus* AFB1	*Pseudomonas* 50 isolates	in vitro	inhibition of AFB1 production in 77% of strains on solid PDA medium mycelial growth suppression on both PDB and PDA media, to varying degrees *Chlororaphis* isolates 66, 68, and 4 fully inhibited AFB1 production on both media types	[127]

**Table 11:** Botanical agents with antifungal and antitoxic properties against *Aspergillus* fungi and aflatoxins.

Aflatoxin and/or Fungus	Type of Botanical Agent	Model	Effects	References
AFB1	Coumarin and chlorophyllin	Rats in vivo	restoration in normal serum biochemical parameters alleviation of histopathological changes in the liver, pancreas, and kidneys improvement in rat growth efficiency	[149]
AFB1 AFB2 AFG1 AFG2	Jatropha pomace extract; jojoba oil; jojoba pomace extract; jatropha oil	in vitro	significant reduction in aflatoxin levels by jatropha pomace extract reduction of AFB1 levels by jojoba oil, jojoba pomace extract, and jatropha oil	[148]
AFB1	Ginger extract	HepG2 cells in vitro; rats in vivo	inhibition of ROS production, DNA strand breaks, and cytotoxicity regulation of the Nrf2/HO-1 pathway reduction in lipid peroxidation increase in antioxidant enzyme activity	[150]
AFs	Banana peel powder	Rats in vivo and in vitro	adsorption of aflatoxins reduction in aflatoxin-induced liver and kidney damage	[147]
AFB1 AFG1	Cape golden berry	Rats in vivo	improvement in body weight increase in nutritional efficiency and weight gain improvement in kidney and liver function, including liver enzymes improvement in tumor markers restoration of lipid, total protein, albumin, globulin, and iron levels to reference values	[151]
Aflatoxins	Date palm (*Phoenix dactylifera*) seeds	Chickens in vivo	alleviation of nephritis and histopathological changes in the intestines reduction in liver and small intestine weight regulation of biochemical parameters of the liver and kidneys dose-dependent protective effect, with better results from 2% than 4% *Phoenix dactylifera* seed supplementation	[152]
AFB1	Black tea and/or curcumin	Rats in vivo	reduction in liver and kidney dysfunction increase in antioxidant glutathione levels reduction in lipid peroxide levels reduction in creatinine, urea, and uric acid levels	[153]
AFB1	Purple waxy corn extract	HepG2 cells in vitro	increase in cell viability reduction in ROS and micronuclei production increase in antioxidant enzyme activity (glutathione reductase, GPx, GST)	[154]
AFB1	Curcumin	Ducks in vivo	protection of the ileum against morphological damage and inflammation reduction in AFB1 DNA adducts in plasma reduction in oxidative stress	[155]
Aflatoxins	Lycopene and silymarin	Ducks in vivo	decreased liver and kidney parameters and MDA increase in antioxidant parameters (TAC, GST, and CAT) significant decrease in alanine aminotransferase, gamma-glutamyltransferase, and ALP (alkaline phosphatase) enzyme activity decrease in total protein and albumin concentration in blood serum increase in catalase activity	[156]
AFB1	Artichoke leaf extract	Rats in vivo	regulation of lipid profile, glucose and insulin levels, homeostasis model assessment of insulin resistance, TNF-α (tumor necrosis factor alpha), and IDO (indoleamine-pyrrole 2,3-dioxygenase) decrease in AChE (acetylcholinesterase) and monoamine oxidase activity increase in homeostasis model assessment of β-cell function, TIMP3 (tissue inhibitor metallopeptidase 3) activity	[157]
AFB1	Curcumin	Ducks in vivo	improvement in animal growth strengthening of spleen immune functions reduction in spleen damage and inflammation activation of Nrf2 signaling pathways increase in expression of related antioxidant enzymes inhibition of NF-kB (nuclear factor kappa B) signaling pathway	[158]
Aflatoxins	Barley microgreen	Rats in vivo	reduction in oxidative stress increase in sperm count reduction in morphological abnormalities, seminiferous tubule degeneration, and chromosomal aberrations	[159]
*A. parasiticus* aflatoxins	Essential oils: lavandin grosso and abrial, *Origanum virens,* *Rosmarinus officinalis*; Phenolic acids: caffeic, chlorogenic, ferulic, p-coumaric	in vitro	complete inhibition of fungal growth by *O. virens* essential oil and lavandin (grosso and abrial) essential oil significant reduction in mycelial weight by all essential oils and phenolic acids strong inhibition of aflatoxin synthesis in all cases (except lavandin abrial) complete inhibition of aflatoxin production by all phenolic acids at 20 mM concentration	[133]
AFB1	Curcumin	Mice in vivo	reduction in weight loss reduction in pyroptosis frequency interleukin (IL)-1β and IL-18 release liver damage and dysfunction and AFB1-DNA adducts accumulation in the liver inhibition of oxidative stress and enhancement of phase II detoxification via GST increase in Nrf2 expression and associated antioxidant molecules (SOD, CAT, HO-1, NQO1), GST, GSH, GSS (glutathione synthetase), GCLC, GCLM (glutamate–cysteine ligase modifier subunit)	[160]
AFB1	Curcumin	Mice in vivo	modulation of Keap1–Nrf2 pathway inhibition of Bax/Bcl-2–Cyt-c (cytochrome c) pathway improvement in renal and biochemical parameters counteraction of pathological changes and regulation of genes and proteins involved in oxidative stress and apoptosis	[161]
AFB1	Quercetin	Bovine fetal hepatocyte-derived cells (BFH12) in vitro	elimination of AFB1 cytotoxicity dose-dependent reduction in AFM1 levels in the cell medium inhibition of GSTA1 (glutathione S-transferase A1) expression significant transcriptional changes for SOD1 and NQO1 at 20 and 30 µM quercetin, respectively dose-dependent reduction of SOD2 levels	[162]
AFB1 AFB2	Vegetable biocholine	Pigs in vivo	increase in efficiency reduction in total protein and globulin levels lowering of TBARS (thiobarbituric acid reactive substances) levels increase in GST activity reduction in liver enlargement	[163]
*A. parasiticus—*15, 16, 24 *A. favus—*18; aflatoxins	*Halimeda opuntia* extract*;* *Turbinaria decurrens* extract*;* *Jania rubens* extract	in vitro	antifungal effect of *H. opuntia* extract against *A. parasiticus—*24 and *A. favus—*18 antifungal activity of *T. decurrens* extract against *A. favus—*18 fungicidal activity of the *J. rubens* extract against *A. parasiticus*—16 and *A. flavus*—18 effective elimination of *A. parasiticus* toxins (16 and 24) by of *H. opuntia* aqueous extract 100% detoxification of AFG1 and AFG2 in: *A. parasiticus* isolates 15, 16, and 24 by *T. decurrens* extract removal of aflatoxins (except AFG2) from *A. parasiticus* isolates 15 and 16 100% detoxification of AFG1 and AFG2 in *A. flavus*—18 and *A. parasiticus—*24 isolates by *J. rubens* extract	[18]
AFB1	Marjoram essential oil	Rabbits in vivo	increase in rabbit growth increase in antioxidant enzyme activity (CAT and GSH) reduction in inflammatory response markers and AFB1 remnants in the liver	[164]
AFB1	Morin	Rats in vivo	significant reduction in serum levels of AST, ALP, LDH, GGT (gamma-glutamyl transferase), CK (creatine kinase), CK-MB (creatine kinase–myocardial band), 8-OHdG (8-hydroxy-2′-deoxyguanosine), IL-1β, IL-6, and TNF-α at 30 mg/kg body weight significant changes in hepatic and cardiac parameters (MDA, GSH, GPx, SOD, and CAT) at 30 mg/kg	[165]
AFB1	Aqueous extracts of *H. bacciferum* (HB), *O. dhofarense* (OD), and *Z. multiflora* (ZM)	Mice in vitro, in vivo	AFB1 degradation: 95% by HB, 93% by OD, and 92% by ZM prevention of weight loss mitigation of oxidative stress, indicated by increased GSH, TAC, and reduced 8-OHdG reduction in liver damage induced by AFB1 exposure	[166]
*A. flavus* AFs	Pumpkin seed oil (PSO); pumpkin seed oil nanoparticles (PSO-NPs)	in vitro	minimum inhibitory concentrations (MICs): 6.25 μL/mL for PSO and 1.5 μL/mL for PSO-NP reduction in AF levels: PSO — up to 66.6%, and PSO-NP — up to 32.2%	[167]

## Abbreviations

The following abbreviations are used in this manuscript:

8-OHdG	8-hydroxy-2′-deoxyguanosine
AChE	acetylcholinesterase
AFB1	aflatoxin B1
AFB2	aflatoxin B2
AFB2a-Arg	aflatoxin B2a–arginine adduct
AFG1	aflatoxin G1
AFG2	aflatoxin G2
AFM1	aflatoxin M1
AFM1-OVA	aflatoxin M1–ovalbumin conjugate
AFs	aflatoxins
AI	artificial intelligence
AKT	protein kinase B
ALP	alkaline phosphatase
AP	active packaging
APA	AF-producing ability
AST	aspartate aminotransferase
ATR-FTIR	attenuated total reflectance–Fourier transform infrared spectroscopy
AuNP	gold nanoparticles
Avi-tag	avidin tag
Bax	Bcl-2-associated X protein
Bcl-2	B-cell lymphoma 2
BIS	Bureau of Indian Standards
CAM	coconut agar medium
CAP	cold atmospheric plasma
CAT	catalase
CHAO50	chao1 richness estimator
CK	creatine kinase
CK-MB	creatine kinase myocardial band
CMA	coconut milk agar
CRISPR-Cas9	clustered regularly interspaced short palindromic repeats—CRISPR-associated protein 9
CS	chitosan
Cyt-c	cytochrome c
EC	European Commission
ELISA	enzyme-linked immunosorbent assay
ERK1/2	extracellular signal-regulated kinases 1 and 2
EU	European Union
FAO	Food and Agriculture Organization
FDA	Food and Drug Administration
GCLC	glutamate–cysteine ligase catalytic subunit
GCLM	glutamate–cysteine ligase modifier subunit
GC-MS	gas chromatography–mass spectrometry
Gd-MOF/USPIO	gadolinium-based metal–organic framework/ultrasmall superparamagnetic iron oxide
GGT	gamma-glutamyl transferase
GM	genetically modified
GPx	glutathione peroxidase
GSH	glutathione
GSS	glutathione synthetase
GSTA1	glutathione S-transferase A1
H/L stress ratio	heterophil-to-lymphocyte stress ratio
HB	*H. bacciferum*
HIGS	host-induced gene silencing
HO-1	heme oxygenase 1
HPLC	high-performance liquid chromatography
HPLC-HRMS	high-performance liquid chromatography–high resolution mass spectrometry
HPLC-MS/MS	high-performance liquid chromatography–tandem mass spectrometry
IARC	International Agency for Research on Cancer
IDO	indoleamine-pyrrole 2,3-dioxygenase
IgG	immunoglobulin G
IL-1β	interleukin 1 beta
IoT	Internet of Things
IR	infrared
JNK	c-Jun N-terminal kinase
Keap1	Kelch-like ECH-associated protein 1
kGy	kilogray
LC-MS/MS	liquid chromatography–tandem mass spectrometry
LDH	lactate dehydrogenase
LOD	limit of detection
mAb	monoclonal antibody
MDA	malondialdehyde
MIC	minimum inhibitory concentration
ML	machine learning
MRS	magnetic relaxation switching
MWCNTs	multi-walled carbon nanotubes
NF-kB	nuclear factor kappa B
NIR	near-infrared
NQO1	NAD(P)H quinone dehydrogenase 1
Nrf2	nuclear factor erythroid 2-related factor 2
OD	*O. dhofarense*
p-AKT	phosphorylated AKT
PCR	polymerase chain reaction
PDA medium	potato dextrose agar
PDB medium	potato dextrose broth
PD-IPCR	proximity-dependent immuno-PCR
PI3K	phosphoinositide 3-kinase
POCT	point-of-care testing
POF	plastic optical fiber
ppb	parts per billion
PSO	pumpkin seed oil
PSO-NP	pumpkin seed oil nanoparticles
PTEN	phosphatase and tensin homologue
qPCR	quantitative PCR
QuEChERS	quick, easy, cheap, effective, rugged, and safe
RNAi	RNA interference
SMILE	smartphone-powered mobile microfluidic lab-on-fiber device
SMRs	superparamagnetic nanoparticles
SOD	superoxide dismutase
SPCE	screen-printed carbon electrode
TAC	total antioxidant capacity
TBARS	thiobarbituric acid reactive substances
TIMP3	tissue inhibitor metallopeptidase 3
TNF-α	tumor necrosis factor alpha
UHPLC-ESI-QTOF	ultrahigh-performance liquid chromatography–electrospray ionization–quadrupole time-of-flight mass spectrometry
UV light	ultraviolet light
UVC	ultraviolet light, C band
UV-vis–NIR	ultraviolet visible–near infrared
vis	visible
WHO	World Health Organization
YES	yeast extract sucrose
ZM	*Z. multiflora*
Zr-LMOF@Cotton	zirconium-based luminescent metal–organic framework on cotton

## References

[1] Kumar P., Mahato D.K., Kamle M., Mohanta T.K., Kang S.G. (2016). Aflatoxins: A global concern for food safety, human health and their management. Front. Microbiol..

[2] Dai C., Tian E., Hao Z., Tang S., Wang Z., Sharma G., Jiang H., Shen J. (2022). Aflatoxin B1 toxicity and protective effects of curcumin: Molecular mechanisms and clinical implications. Antioxidants.

[3] Ahmad M.M., Qamar F., Saifi M., Abdin M.Z. (2022). Natural inhibitors: A sustainable way to combat aflatoxins. Front. Microbiol..

[4] International Agency for Research on Cancer (IARC) (2012). Aflatoxins. IARC Monographs on the Evaluation of Carcinogenic Risks to Humans.

[5] Ofori-Attah E., Hashimoto M., Oki M., Kadowaki D. (2024). Therapeutic effect of natural products and dietary supplements on aflatoxin-induced nephropathy. Int. J. Mol. Sci..

[6] Faraji H., Yazdi F.T., Razmi N. (2022). The influence of ultraviolet radiation on aflatoxin producing *Aspergillus* species isolated from Iranian rice. Toxicol. Rep..

[7] Romero-Sánchez I., Gracia-Lor E., Madrid-Albarrán Y. (2024). Aflatoxin detoxification by thermal cooking treatment and evaluation of in vitro bioaccessibility from white and brown rice. Food Chem..

[8] Nazareth T.d.M., Soriano Pérez E., Luz C., Meca G., Quiles J.M. (2024). Comprehensive review of aflatoxin and ochratoxin A dynamics: Emergence, toxicological impact, and advanced control strategies. Foods.

[9] Yang X., Zhang Q., Chen Z.-Y., Liu H., Li P. (2017). Investigation of *Pseudomonas fluorescens* strain 3JW1 on preventing and reducing aflatoxin contaminations in peanuts. PLoS ONE.

[10] Popescu R.G., Rădulescu A.L., Georgescu S.E., Dinischiotu A. (2022). Aflatoxins in feed: Types, metabolism, health consequences in swine and mitigation strategies. Toxins.

[11] Mahmoud Y.A., Elkaliny N.E., Darwish O.A., Ashraf Y., Ebrahim R.A., Das S.P., Yahya G. (2025). Comprehensive review for aflatoxin detoxification with special attention to cold plasma treatment. Mycotoxin Res..

[12] Jalili C., Ranjbar Shamsi R., Amiri B., Kakebaraie S., Jalili F., Nasta T.Z. (2024). Genotoxic and cytotoxic effects of aflatoxin on the reproductive system: Focus on cell cycle dynamics and apoptosis in testicular tissue. Toxicology.

[13] FAO, WHO (2017). Aflatoxins. Evaluation of Certain Contaminants in Food.

[14] Navale V., Vamkudoth K.R., Ajmera S., Dhuri V. (2021). *Aspergillus* derived mycotoxins in food and the environment: Prevalence, detection, and toxicity. Toxicol. Rep..

[15] Miklós G., Angeli C., Ambrus Á., Nagy A., Kardos V., Zentai A., Kerekes K., Farkas Z., Jóźwiak Á., Bartók T. (2020). Detection of aflatoxins in different matrices and food-chain positions. Front. Microbiol..

[16] Rushing B.R., Selim M.I. (2017). Adduction to arginine detoxifies aflatoxin B1 by eliminating genotoxicity and altering in vitro toxicokinetic profiles. Oncotarget.

[17] El-Shanshoury A.R., Metwally M.A., El-Sabbagh S.M., Emara H.A., Saba H.E. (2022). Biocontrol of *Aspergillus flavus* producing aflatoxin B1 by *Streptomyces exfoliates*. Egypt. J. Bot..

[18] Farghl A.A.M., El-Sheekh M.M., El-Shahir A.A. (2023). Seaweed extracts as biological control of aflatoxins produced by *Aspergillus parasiticus* and *Aspergillus flavus*. Egypt. J. Biol. Pest Control.

[19] Hojjati M., Shahbazi S., Askari H., Makari M. (2023). Use of X-Irradiations in reducing the waste of aflatoxin-contaminated pistachios and evaluation of the physicochemical properties of the irradiated product. Foods.

[20] García-Ramón D.F., Cornelio-Santiago H.P., Norabuena E., Sumarriva L., Álvarez-Chancasanampa H., Vega M.N., Sotelo-Méndez A., Espinoza-Espinoza L.A., Pantoja-Tirado L.R., Gonzales-Agama S.H. (2025). Effective novel and conventional technologies for decontamination of aflatoxin B_1_ in foods: A review. Mycotoxin Res..

[21] European Commission (2006). Commission Regulation No 1881/2006: Setting maximum levels for certain contaminants in foodstuffs. Off. J. Eur. Union.

[22] Missouri Department of Agriculture (MDA) Aflatoxin Information. https://agriculture.mo.gov/plants/feed/aflatoxin.php.

[23] Hao W.H., Li A., Wang J., An G., Guan S. (2022). Mycotoxin contamination of feeds and raw materials in China in year 2021. Front. Vet. Sci..

[24] Brasil Ministério da Saúde (2011). Agência Nacional de Vigilância Sanitária. Resolução RDC N. 7, de 18 de fevereiro de 2011. Aprova o Regulamento Técnico sobre limites máximos tolerados (LMT) para micotoxinas em alimentos; Seção 1. D. Of. Da Repúb. Fed. Do Bras..

[25] Thakur S., Singh R.K., De P.S., Dey A. (2022). Aflatoxins in feeds: Issues and concerns with safe food production. Indian J. Anim. Health.

[26] Sowmya K.L., Ramalingappa B. (2024). Rapid detection of aflatoxin production by *Aspergillus flavus* using coconut agar medium. Food Sci. Nutr. Technol..

[27] Ronoh P.K., Toroitich F.J., Makonde H.M., Lelmen E.K., Obonyo M.A. (2024). Reliability of the chemical, metabolic, and molecular methods in discriminating aflatoxigenic from non-aflatoxigenic *Aspergillus* isolates. Microbe.

[28] Alameri M.M., Kong S.-Y., Aljaafari M.N., Ali H.A., Eid K., Sallagi M.A., Cheng W.-H., Abushelaibi A., Lim S.-H.E., Loh J.-Y. (2023). Aflatoxin contamination: An overview on health issues, detection and management strategies. Toxins.

[29] Sudini H., Srilakshmi P., Kumar K.V.K., Njoroge S.M.C., Osiru M., Seetha A., Waliyar F. (2015). Detection of aflatoxigenic *Aspergillus* strains by cultural and molecular methods: A critical review. Afr. J. Microbiol. Res..

[30] Akinola S.A., Ateba C.N., Mwanza M. (2019). Polyphasic assessment of aflatoxin production potential in selected *Aspergilli*. Toxins.

[31] Bharose A.A., Hajare S.T., Narayanrao D.R., Gajera H.G., Prajapati H.K., Singh S.C., Upadhye V. (2024). Whole genome sequencing and annotation of *Aspergillus flavus* JAM-JKB-B HA-GG20. Sci. Rep..

[32] Ortega S.F., Siciliano I., Prencipe S., Gullino M.L., Spadaro D. (2020). Development of PCR, LAMP, and qPCR assays for the detection of aflatoxigenic strains of *Aspergillus flavus* and *A. parasiticus* in hazelnut. Toxins.

[33] Mahmoud M.A. (2015). Detection of *Aspergillus flavus* in stored peanuts using real-time PCR and the expression of aflatoxin genes in toxigenic and atoxigenic *A. flavus* isolates. Foodborne Pathog. Dis..

[34] Bintvihok A., Treebonmuang S., Srisakwattana K., Nuanchun W., Patthanachai K., Usawang S. (2016). A rapid and sensitive detection of aflatoxin-producing fungus using an optimized polymerase chain reaction (PCR). Toxicol. Res..

[35] Xu L., Qu W., Hao X., Fang M., Yang Q., Li Y., Gong Z., Li P. (2024). Immunochromatographic strip based on tetrahedral DNA immunoprobe for the detection of aflatoxin B_1_ in rice bran oil. Foods.

[36] He K., Bu T., Zhao S., Bai F., Zhang M., Tian Y., Sun X., Dong M., Wang L. (2021). Well-orientation strategy for direct binding of antibodies: Development of the immunochromatographic test using the antigen modified Fe_2_O_3_ nanoprobes for sensitive detection of aflatoxin B1. Food Chem..

[37] Sun J., Li M., Xing F., Wang H., Zhang Y., Sun X. (2022). Novel dual immunochromatographic test strip based on double antibodies and biotin-streptavidin system for simultaneous sensitive detection of aflatoxin M1 and ochratoxin A in milk. Food Chem..

[38] Zhuo Y., Xu W., Chen Y., Long F. (2023). Rapid and sensitive point-of-need aflatoxin B1 testing in feedstuffs using a smartphone-powered mobile microfluidic lab-on-fiber device. J. Hazard. Mater..

[39] Liu J.-W., Lu C.C., Liu B.-H., Yu F.-Y. (2016). Development of novel monoclonal antibodies-based ultrasensitive enzyme-linked immunosorbent assay and rapid immunochromatographic strip for aflatoxin B1 detection. Food Control.

[40] Zhang X., Wen K., Wang Z., Jiang H., Beier R.C., Shen J. (2016). An ultra-sensitive monoclonal antibody-based fluorescent microsphere immunochromatographic test strip assay for detecting aflatoxin M_1_ in milk. Food Control.

[41] Wu C., Liu D., Peng T., Shan S., Zhang G., Xiong Y., Lai W. (2016). Development of a one-step immunochromatographic assay with two cutoff values of aflatoxin M1. Food Control.

[42] Shao Y., Duan H., Guo L., Leng Y., Lai W., Xiong Y. (2018). Quantum dot nanobead-based multiplexed immunochromatographic assay for simultaneous detection of aflatoxin B1 and zearalenone. Anal. Chim. Acta.

[43] Sojinrin T., Liu K., Wang K., Cui D., Byrne H.J., Curtin J.F., Tian F. (2019). Developing gold nanoparticles-conjugated aflatoxin B1 antifungal strips. Int. J. Mol. Sci..

[44] Pietschmann J., Spiegel H., Krause H.-J., Schillberg S., Schröper F. (2020). Sensitive aflatoxin B1 detection using nanoparticle-based competitive magnetic immunodetection. Toxins.

[45] Wu S.-W., Ko J.-L., Liu B.-H., Yu F.-Y. (2020). A sensitive two-analyte immunochromatographic strip for simultaneously detecting aflatoxin M1 and chloramphenicol in milk. Toxins.

[46] Jiang S., Zhang L., Li J., Ouyang H., Fu Z. (2021). Pressure/colorimetric dual-readout immunochromatographic test strip for point-of-care testing of aflatoxin B1. Talanta.

[47] Zhao X., Jin X., Lin Z., Guo Q., Liu B., Yuan Y., Yue T., Zhao X. (2021). Simultaneous rapid detection of aflatoxin B1 and ochratoxin A in spices using lateral flow immuno-chromatographic assay. Foods.

[48] Peltomaa R., Abbas A., Yli-Mattila T., Lamminmäki U. (2022). Single-step noncompetitive immunocomplex immunoassay for rapid aflatoxin detection. Food Chem..

[49] Wang Y., Liu F., Zhou X., Liu M., Zang H., Liu X., Shan A., Feng X. (2022). Alleviation of oral exposure to aflatoxin B1-induced renal dysfunction, oxidative stress, and cell apoptosis in mice kidney by curcumin. Antioxidants.

[50] Zhang J., Li X., Xie J., Huang Z. (2023). Rapid and simultaneous detection of aflatoxin B1, zearalenone, and T-2 toxin in medicinal and edible food using gold immunochromatographic test strip. Foods.

[51] Wang X., Sun T., Shen W., Liu M., Liu W., Zuo H., Zhang Y., Geng L., Wang W., Shao C. (2023). A lateral flow immunochromatographic assay based on nanobody-oriented coupling strategy for aflatoxin B1 detection. Sens. Actuators B Chem..

[52] Liu S., Jiang S., Yao Z., Liu M. (2023). Aflatoxin detection technologies: Recent advances and future prospects. Environ. Sci. Pollut. Res. Int..

[53] Li Y., Zhang Y., Han J., Chu P.K., Feng J., Dong Y. (2017). A sensitive non-enzymatic immunosensor composed of silver nanoflowers for squamous cell carcinoma antigen. RSC Adv..

[54] Pal T., Aditya S., Mathai T., Mukherji S. (2023). Polyaniline-coated plastic optic fiber biosensor for detection of aflatoxin B1 in nuts, cereals, beverages, and body fluids. Sens. Actuators B Chem..

[55] Lu Y., Chen R., Dong Y., Zhao W., Ruan S., Yang W., Chen Y., Wang C. (2023). Magnetic relaxation switching immunoassay based on “limited-magnitude” particles for sensitive quantification of aflatoxin B1. Anal. Chim. Acta.

[56] Guo G., Wang T., Liu Z., Liu X., Li T., Chen Y., Fan J., Bukye E., Huang X., Song L. (2023). A self-assembled DNA double-crossover-based fluorescent aptasensor for highly sensitivity and selectivity in the simultaneous detection of aflatoxin M_1_ and aflatoxin B_1_. Talanta.

[57] Uludag Y., Esen E., Kokturk G., Ozer H., Muhammad T., Olcer Z., Basegmez H.I.O., Simsek S., Barut S., Gok M.Y. (2016). Lab-on-a-chip based biosensor for the real-time detection of aflatoxin. Talanta.

[58] Azri F., Sukor R., Selamat J., Bakar F.A., Yusof N., Hajian R. (2018). Electrochemical immunosensor for detection of aflatoxin B1 based on indirect competitive ELISA. Toxins.

[59] Abera B.D., Falco A., Ibba P., Cantarella G., Petti L., Lugli P. (2019). Development of flexible dispense-printed electrochemical immunosensor for aflatoxin M1 detection in milk. Sensors.

[60] Ren X., Zhang Q., Wu W., Yan T., Tang X., Zhang W., Yu L., Li P. (2019). Anti-idiotypic nanobody-phage display-mediated real-time immuno-PCR for sensitive, simultaneous and quantitative detection of total aflatoxins and zearalenone in grains. Food Chem..

[61] Hu D., Xiao S., Guo Q., Yue R., Geng D., Ji D. (2021). Luminescence method for detection of aflatoxin B1 using ATP-releasing nucleotides. RSC Adv..

[62] Zhang S., Li Z., An J., Yang Y., Tang X. (2021). Identification of aflatoxin B1 in peanut using near-infrared spectroscopy combined with naive bayes classifier. Spectrosc. Lett..

[63] Zhao L., Mao J., Hu L., Zhang S., Yang X. (2021). Self-replicating catalyzed hairpin assembly for rapid aflatoxin B1 detection. Anal. Methods.

[64] Dai S., Xing K., Jiao Y., Yu S., Yang X., Yao L., Jia P., Cheng Y., Xu Z. (2024). A novel magnetic resonance tuning-magnetic relaxation switching sensor based on Gd-MOF/USPIO assembly for sensitive and convenient aflatoxin B1 detection. Food Chem..

[65] Huang S., Song X., Wang S., Liu H., Xiong C., Wang S., Zhang X., Chen M.M. (2024). Portable dual-mode paper chips for highly sensitive and rapid determination of aflatoxin B_1_ via an aptamer-gated MOFs. Food Chem..

[66] Wu W., Bai Y., Zhao T., Liang M., Hu X., Wang D., Tang X., Yu L., Zhang Q., Li P. (2023). Intelligent electrochemical point-of-care test method with interface control based on DNA pyramids: Aflatoxin B1 detection in food and the environment. Foods.

[67] Li Y., Li Z., Jia B., Tu Z., Zeng J., Pang J., Ren W., Huang Z., He B., Wang Z. (2024). Detection of AFB_1_ by immunochromatographic test strips based on double-probe signal amplification with nanobody and biotin–streptavidin system. Foods.

[68] Zhao H., Chen X., Shen C., Qu B. (2016). Determination of 16 mycotoxins in vegetable oils using a QuEChERS method combined with high-performance liquid chromatography-tandem mass spectrometry. Food Addit. Contam. A.

[69] Sharmili K., Jinap S., Sukor R. (2016). Development, optimization and validation of QuEChERS based liquid chromatography tandem mass spectrometry method for determination of multimycotoxin in vegetable oil. Food Control.

[70] Lijalem Y.G., Gab-Allah M.A., Yu H., Choi K., Kim B. (2024). Development of isotope dilution-ultrahigh-performance liquid chromatography-tandem mass spectrometry for the accurate determination of aflatoxins in grains. J. Food Compost. Anal..

[71] Zhao Y., Huang J., Ma L., Wang F. (2017). Development and validation of a simple and fast method for simultaneous determination of aflatoxin B1 and sterigmatocystin in grains. Food Chem..

[72] Shuib N.S., Makahleh A., Salhimi S.M., Saad B. (2017). Determination of aflatoxin M1 in milk and dairy products using high performance liquid chromatography-fluorescence with post column photochemical derivatization. J. Chromatogr. A.

[73] Campone L., Piccinelli A.L., Celano R., Pagano I., Di Sanzo R., Carabetta S., Russo M., Rastrelli L. (2018). Occurrence of aflatoxin M1 in milk samples from Italy analysed by online-SPE UHPLC-MS/MS. Nat. Prod. Res..

[74] Paschoal F.N., De Azevedo Silva D., Von Sperling De Souza R., De Oliveira M.S., Pereira D.A.A., De Souza S.V.C. (2017). A rapid single-extraction method for the simultaneous determination of aflatoxins B1, B2, G1, G2, fumonisin B1, and zearalenone in corn meal by ultra-performance liquid chromatography tandem mass spectrometry. Food Anal. Methods.

[75] Mao J., Zheng N., Wen F., Guo L., Fu C., Ouyang H., Zhong L., Wang J., Lei S. (2018). Multi-mycotoxins analysis in raw milk by ultra-high performance liquid chromatography coupled to quadrupole orbitrap mass spectrometry. Food Control.

[76] Flores-Flores M.E., González-Peñas E. (2017). An LC–MS/MS method for multi-mycotoxin quantification in cow milk. Food Chem..

[77] Sartori A.V., De Moraes M.H.P., Dos Santos R.P., Souza Y.P., Da Nóbrega A.W. (2017). Determination of mycotoxins in cereal-based porridge destined for infant consumption by ultra-high performance liquid chromatography-tandem mass spectrometry. Food Anal. Methods.

[78] Huertas-Pérez J.F., Arroyo-Manzanares N., Hitzler D., Castro-Guerrero F.G., Gámiz-Gracia L., García-Campaña A.M. (2018). Simple determination of aflatoxins in rice by ultra-high performance liquid chromatography coupled to chemical post-column derivatization and fluorescence detection. Food Chem..

[79] Zareshahrabadi Z., Bahmyari R., Nouraei H., Khodadadi H., Mehryar P., Asadian F., Zomorodian K. (2020). Detection of aflatoxin and ochratoxin A in spices by high-performance liquid chromatography. J. Food Qual..

[80] Algammal A.M., Elsayed M.E., Hashem H.R., Ramadan H., Sheraba N.S., El-Diasty E.M., Abbas S.M., Hetta H.F. (2021). Molecular and HPLC-based approaches for detection of aflatoxin B1 and ochratoxin A released from toxigenic *Aspergillus* species in processed meat. BMC Microbiol..

[81] Salisu B., Anua S., Isha W., Mazlan N. (2021). Development and validation of quantitative thin layer chromatographic technique for determination of total aflatoxins in poultry feed and food grains without sample clean-up. J. Adv. Vet. Anim. Res..

[82] Al-Ghouti M.A., AlHusaini A., Abu-Dieyeh M.H., Abd Elkhabeer M., Alam M.M. (2022). Determination of aflatoxins in coffee by means of ultra-high performance liquid chromatography-fluorescence detector and fungi isolation. Int. J. Environ. Anal. Chem..

[83] Smeesters L. (2024). Fluorescence spectroscopy enhancing aflatoxin detection in solid food products: From laboratory setup towards handheld sensing units. Biophotonics Point Care III.

[84] Jiang H., Zhao Y., Li J., Zhao M., Deng J., Bai X. (2024). Quantitative detection of aflatoxin B1 in peanuts using Raman spectra and multivariate analysis methods. Spectrochim. Acta A Mol. Biomol. Spectrosc..

[85] Yao S., Miyagusuku-Cruzado G., West M., Nwosu V., Dowd E., Fountain J., Giusti M.M., Rodriguez-Saona L.E. (2024). Nondestructive and rapid screening of aflatoxin-contaminated single peanut kernels using field-portable spectroscopy instruments (FT-IR and Raman). Foods.

[86] Smeesters L., Meulebroeck W., Raeymaekers S., Thienpont H. (2015). Optical detection of aflatoxins in maize using one- and two-photon induced fluorescence spectroscopy. Food Control.

[87] Magnus I., Abbasi F., Thienpont H., Smeesters L. (2024). Laser-induced fluorescence spectroscopy enhancing pistachio nut quality screening. Food Control.

[88] Durmuş E., Güneş A., Kalkan H. (2017). Detection of aflatoxin and surface mould contaminated figs by using Fourier transform Near-Infrared Reflectance spectroscopy. J. Sci. Food Agric..

[89] Lai W., Zeng Q., Tang J., Zhang M., Tang D. (2018). A conventional chemical reaction for use in an unconventional assay: A colorimetric immunoassay for aflatoxin B1 by using enzyme-responsive just-in-time generation of a MnO_2_ based nanocatalyst. Microchim. Acta.

[90] Jaiswal P., Jha S.N., Kaur J., Borah A., Ramya H.G. (2018). Detection of aflatoxin M1 in milk using spectroscopy and multivariate analyses. Food Chem..

[91] Cheng X., Vella A., Stasiewicz M.J. (2019). Classification of aflatoxin contaminated single corn kernels by ultraviolet to near infrared spectroscopy. Food Control.

[92] Shen F., Wu Q., Shao X., Zhang Q. (2018). Non-destructive and rapid evaluation of aflatoxins in brown rice by using near-infrared and mid-infrared spectroscopic techniques. J. Food Sci. Technol..

[93] Wu Q., Xu H. (2019). Application of multiplexing fiber optic laser induced fluorescence spectroscopy for detection of aflatoxin B1 contaminated pistachio kernels. Food Chem..

[94] Bertani F.R., Businaro L., Gambacorta L., Mencattini A., Brenda D., Di Giuseppe D., De Ninno A., Solfrizzo M., Martinelli E., Gerardino A. (2020). Optical detection of aflatoxins B in grained almonds using fluorescence spectroscopy and machine learning algorithms. Food Control.

[95] Putthang R., Sirisomboon P., Sirisomboon C.D. (2019). Shortwave near-infrared spectroscopy for rapid detection of aflatoxin B1 contamination in polished rice. J. Food Prot..

[96] Tao F., Yao H., Zhu F., Hruska Z., Liu Y., Rajasekaran K., Bhatnagar D. (2019). A rapid and nondestructive method for simultaneous determination of aflatoxigenic fungus and aflatoxin contamination on corn kernels. J. Agric. Food Chem..

[97] Liu W., Zhao P., Wu C., Liu C., Yang J., Zheng L. (2019). Rapid determination of aflatoxin B1 concentration in soybean oil using terahertz spectroscopy with chemometric methods. Food Chem..

[98] Wu Q., Xu H. (2020). Design and development of an on-line fluorescence spectroscopy system for detection of aflatoxin in pistachio nuts. Postharvest Biol. Technol..

[99] Zhongzhi H., Limiao D. (2020). Aflatoxin contaminated degree detection by hyperspectral data using band index. Food Chem. Toxicol..

[100] Zheng S.-Y., Wei Z.-S., Li S., Zhang S.-J., Xie C.-F., Yao D.-S., Liu D.-L. (2020). Near-infrared reflectance spectroscopy-based fast versicolorin A detection in maize for early aflatoxin warning and safety sorting. Food Chem..

[101] Chen M., He X., Pang Y., Shen F., Fang Y., Hu Q. (2021). Laser induced fluorescence spectroscopy for detection of aflatoxin B1 contamination in peanut oil. J. Food Meas. Charact..

[102] Jha S.N., Jaiswal P., Kaur J., Ramya H.G. (2021). Rapid detection and quantification of aflatoxin B1 in milk using Fourier transform infrared spectroscopy. J. Inst. Eng. A.

[103] Tang W., Qi Y., Li Z. (2021). A portable, cost-effective and user-friendly instrument for colorimetric enzyme-linked immunosorbent assay and rapid detection of aflatoxin B1. Foods.

[104] Chavez R.A., Cheng X., Herrman T.J., Stasiewicz M.J. (2022). Single kernel aflatoxin and fumonisin contamination distribution and spectral classification in commercial corn. Food Control.

[105] Bartolić D., Mutavdžić D., Carstensen J.M., Stanković S., Nikolić M., Krstović S., Radotić K. (2022). Fluorescence spectroscopy and multispectral imaging for fingerprinting of aflatoxin-B1 contaminated (*Zea mays* L.) seeds: A preliminary study. Sci. Rep..

[106] Liu T., He J., Yao W., Jiang H., Chen Q. (2022). Determination of aflatoxin B1 value in corn based on Fourier transform near-infrared spectroscopy: Comparison of optimization effect of characteristic wavelengths. LWT.

[107] Smeesters L., Kuntzel T., Thienpont H., Guilbert L. (2023). Handheld fluorescence spectrometer enabling sensitive aflatoxin detection in maize. Toxins.

[108] Lutz É., Carteri Coradi P. (2022). Applications of new technologies for monitoring and predicting grains quality stored: Sensors, Internet of Things, and Artificial Intelligence. Measurement.

[109] Zhang W., Dou J., Wu Z., Li Q., Wang S., Xu H., Wu W., Sun C. (2022). Application of non-aflatoxigenic *Aspergillus flavus* for the biological control of aflatoxin contamination in China. Toxins.

[110] Nguyen T., Chen X., Ma L., Feng Y. (2024). Mycotoxin biodegradation by *Bacillus* bacteria—A review. Toxins.

[111] Shu X., Wang Y., Zhou Q., Li M., Hu H., Ma Y., Chen X., Ni J., Zhao W., Huang S. (2018). Biological degradation of aflatoxin B_1_ by cell-free extracts of *Bacillus velezensis* DY3108 with broad pH stability and excellent thermostability. Toxins.

[112] Xu L., Ahmed M.F.E., Sangare L., Zhao Y., Selvaraj J.N., Xing F., Wang Y., Yang H., Liu Y. (2017). Novel aflatoxin-degrading enzyme from *Bacillus shackletonii* L7. Toxins.

[113] Zhang L.Y., Liu S., Zhao X.J., Wang N., Jiang X., Xin H.S., Zhang Y.G. (2019). *Lactobacillus rhamnosus* GG modulates gastrointestinal absorption, excretion patterns, and toxicity in Holstein calves fed a single dose of aflatoxin B1. J. Dairy Sci..

[114] Intanoo M., Kongkeitkajorn M.B., Suriyasathaporn W., Phasuk Y., Bernard J.K., Pattarajinda V. (2020). Effect of supplemental *Kluyveromyces marxianus* and *Pichia kudriavzevii* on aflatoxin M1 excretion in milk of lactating dairy cows. Animals.

[115] Lopes D.C., Martins J.H., Lacerda Filho A.F., Melo E.C., Monteiro P.M.B., Queiroz D.M. (2008). Aeration strategy for controlling grain storage based on simulation and on real data acquisition. Comput. Electron. Agric..

[116] Ghanbari R., Aghaee E.M., Rezaie S., Khaniki G.J., Alimohammadi M., Soleimani M., Noorbakhsh F. (2017). The inhibitory effect of lactic acid bacteria on aflatoxin production and expression of *aflR* gene in *Aspergillus parasiticus*. J. Food Saf..

[117] Siahmoshteh F., Hamidi-Esfahani Z., Spadaro D., Shams-Ghahfarokhi M., Razzaghi-Abyaneh M. (2018). Unraveling the mode of antifungal action of *Bacillus subtilis* and *Bacillus amyloliquefaciens* as potential biocontrol agents against aflatoxigenic *Aspergillus parasiticus*. Food Control.

[118] Quattrini M., Bernardi C., Stuknytė M., Masotti F., Passera A., Ricci G., Vallone L., De Noni I., Brasca M., Fortina M.G. (2018). Functional characterization of *Lactobacillus plantarum* ITEM 17215: A potential biocontrol agent of fungi with plant growth promoting traits, able to enhance the nutritional value of cereal products. Food Res. Int..

[119] González Pereyra M.L., Martínez M.P., Petroselli G., Erra Balsells R., Cavaglieri L.R. (2018). Antifungal and aflatoxin-reducing activity of extracellular compounds produced by soil *Bacillus* strains with potential application in agriculture. Food Control.

[120] Yang M., Lu L., Pang J., Hu Y., Guo Q., Li Z., Wu S., Liu H., Wang C. (2019). Biocontrol activity of volatile organic compounds from *Streptomyces alboflavus* TD-1 against *Aspergillus flavus* growth and aflatoxin production. J. Microbiol..

[121] Liu Y., Mao H., Hu C., Tron T., Lin J., Wang J., Sun B. (2020). Molecular docking studies and in vitro degradation of four aflatoxins (AFB_1_, AFB_2_, AFG_1_, and AFG_2_) by a recombinant laccase from *Saccharomyces cerevisiae*. J. Food Sci..

[122] Fouad M., El-desouky T.A. (2020). Anti-toxigenic effect of lactic acid bacteria against *Aspergillus* spp. isolated from wheat grains. Open Microbiol. J..

[123] da Cruz P.O., de Matos C.J., Nascimento Y.M., Tavares J.F., de Souza E.L., Magalhães H.I.F. (2021). Efficacy of potentially probiotic fruit-derived *Lactobacillus fermentum*, L. *paracasei* and L. *plantarum* to remove aflatoxin M1 in vitro. Toxins.

[124] Rämö S., Kahal M., Joutsjoki V. (2022). Aflatoxin B1 binding by lactic acid bacteria in protein-rich plant material fermentation. Appl. Sci..

[125] Kumar V., Bahuguna A., Ramalingam S., Lee J.S., Han S.S., Chun H.S., Kim M. (2022). Aflatoxin reduction and retardation of aflatoxin production by microorganisms in doenjang during a one-year fermentation. J. Fungi.

[126] Chen G., Fang Q., Liao Z., Xu C., Liang Z., Liu T., Zhong Q., Wang L., Fang X., Wang J. (2022). Detoxification of aflatoxin B1 by a potential probiotic *Bacillus amyloliquefaciens* WF2020. Front. Microbiol..

[127] Papp D.A., Kocsubé S., Farkas Z., Szekeres A., Vágvölgyi C., Hamari Z., Varga M. (2024). Aflatoxin B1 control by various *Pseudomonas* isolates. Toxins.

[128] Patras A., Julakanti S., Yannam S.R.R., Bansode M.R., Burns M., Vergne M.J. (2017). Effect of UV irradiation on aflatoxin reduction: A cytotoxicity evaluation study using human hepatoma cell line. Mycotoxin Res..

[129] Domijan A.M., Čermak A.M.M., Vulić A., Bujak I.T., Pavičić I., Pleadin J., Markov K., Mihaljević B. (2019). Cytotoxicity of gamma irradiated aflatoxin B1 and ochratoxin A. J. Environ. Sci. Health B.

[130] Ferreira C.D., Lang G.H., Da Silva Lindemann I., Da Silva Timm N., Hoffmann J.F., Ziegler V., De Oliveira M. (2021). Postharvest UV-C irradiation for fungal control and reduction of mycotoxins in brown, black, and red rice during long-term storage. Food Chem..

[131] Hojnik N., Modic M., Walsh J.L., Žigon D., Javornik U., Plavec J., Žegura B., Filipič M., Cvelbar U. (2021). Unravelling the pathways of air plasma induced aflatoxin B1 degradation and detoxification. J. Hazard. Mater..

[132] Rushing B.R., Selim M.I. (2019). Aflatoxin B1: A review on metabolism, toxicity, occurrence in food, occupational exposure, and detoxification methods. Food Chem. Toxicol..

[133] Lorán S., Carramiñana J.J., Juan T., Ariño A., Herrera M. (2022). Inhibition of *Aspergillus parasiticus* growth and aflatoxins production by natural essential oils and phenolic acids. Toxins.

[134] Zheng N., Zhang H., Li S., Wang J., Liu J., Ren H., Gao Y. (2018). Lactoferrin inhibits aflatoxin B1- and aflatoxin M1-induced cytotoxicity and DNA damage in Caco-2, HEK, Hep-G2, and SK-N-SH cells. Toxicon.

[135] Akbari D.M., Kordbacheh P., Daei Ghazvini R., Moazeni M., Nazemi L., Rezaie S. (2018). Inhibitory effect of vitamin C on *Aspergillus parasiticus* growth and aflatoxin gene expression. Curr. Med. Mycol..

[136] Mahbobinejhad Z., Aminian H., Ebrahimi L., Vahdati K. (2019). Reduction of aflatoxin production by exposing *Aspergillus flavus* to CO_2_. J. Crop Prot..

[137] Li H., Li S., Yang H., Wang Y., Wang J., Zheng N. (2019). L-proline alleviates kidney injury caused by AFB1 and AFM1 through regulating excessive apoptosis of kidney cells. Toxins.

[138] Yu Y., Shi J., Xie B., He Y., Qin Y., Wang D., Shi H., Ke Y., Sun Q. (2020). Detoxification of aflatoxin B1 in corn by chlorine dioxide gas. Food Chem..

[139] Al-Kuhla A.A.M., Ibraheem A.M. (2021). Protective effects of rhino-hepato forte in broiler chickens during aflatoxicosis. Egypt. J. Vet. Sci..

[140] Wu G., San J., Pang H., Du Y., Li W., Zhou X., Yang X., Hu J., Yang J. (2022). Taurine attenuates AFB1-induced liver injury by alleviating oxidative stress and regulating mitochondria-mediated apoptosis. Toxicon.

[141] Liu Y., Wang J., Chang Z., Li S., Zhang Z., Liu S., Wang S., Wei L., Lv Q., Ding K. (2024). SeMet alleviates AFB1-induced oxidative stress and apoptosis in rabbit kidney by regulating Nrf2/Keap1/NQO1 and PI3K/AKT signaling pathways. Ecotoxicol. Environ. Saf..

[142] Yang D., Zhang S., Cao H., Wu H., Liang Y., Teng C.B., Yu H.F. (2024). Detoxification of aflatoxin B_1_ by phytochemicals in agriculture and food science. J. Agric. Food Chem..

[143] Haque M.A., Wang Y., Shen Z., Li X., Saleemi M.K., He C. (2020). Mycotoxin contamination and control strategy in human, domestic animal and poultry: A review. Microb. Pathog..

[144] Silva B.A., Ferreres F., Malva J.O., Dias A.C.P. (2005). Phytochemical and antioxidant characterization of *Hypericum perforatum* alcoholic extracts. Food Chem..

[145] Brborić J., Klisić A., Kotur-Stevuljević J., Delogu G., Gjorgieva Ackova D., Kostić K., Dettori M.A., Fabbri D., Carta P., Saso L. (2021). Natural and natural-like polyphenol compounds: In vitro antioxidant activity and potential for therapeutic application. Arch. Med. Sci..

[146] Abdel-Razek A.G., Badr A.N., Alharthi S.S., Selim K.A. (2021). Efficacy of bottle gourd seeds’ extracts in chemical hazard reduction secreted as toxigenic fungi metabolites. Toxins.

[147] Ali D., Abdel-Rahman T., Abo-Hagger A., Ahmed M. (2019). In vitro and in vivo assessment of banana peel powder as an aflatoxins biosorbent. Egypt. J. Bot..

[148] Badr A.N., Shehata M.G., Abdel-Razek A.G. (2017). Antioxidant activities and potential impacts to reduce aflatoxins utilizing jojoba and jatropha oils and extracts. Int. J. Pharmacol..

[149] Abdel-Latif M.S., Elmeleigy K.M., Aly T.A.A., Khattab M.S., Mohamed S.M. (2017). Pathological and biochemical evaluation of coumarin and chlorophyllin against aflatoxicosis in rat. Exp. Toxicol. Pathol..

[150] Vipin A.V., Raksha Rao K., Kurrey N.K., Appaiah K.A., Venkateswaran G. (2017). Protective effects of phenolics-rich extract of ginger against aflatoxin B1-induced oxidative stress and hepatotoxicity. Biomed. Pharmacother..

[151] Badr A.N., Naeem M.A. (2019). Protective efficacy using cape-golden berry against pre-carcinogenic aflatoxins induced in rats. Toxicol. Rep..

[152] Abdel-Sattar W.M., Sadek K.M., Elbestawy A.R., Mourad D.M. (2019). The protective role of date palm (*Phoenix dactylifera* seeds) against aflatoxicosis in broiler chickens regarding carcass characteristics, hepatic and renal biochemical function tests and histopathology. J. World’s Poult. Res..

[153] El-Mekkawy H.I., Al-Kahtani M.A., Shati A.A., Alshehri M.A., Al-Doaiss A.A., Elmansi A.A., Ahmed A.E. (2020). Black tea and curcumin synergistically mitigate the hepatotoxicity and nephropathic changes induced by chronic exposure to aflatoxin-B1 in Sprague-Dawley rats. J. Food Biochem..

[154] Singto T., Tassaneeyakul W., Porasuphatana S. (2020). Protective effects of purple waxy corn on aflatoxin B1-induced oxidative stress and micronucleus in HepG2 cells. Indian J. Pharm. Sci..

[155] Jin S., Yang H., Jiao Y., Pang Q., Wang Y., Wang M., Shan A., Feng X. (2021). Dietary curcumin alleviated acute ileum damage of ducks (*Anas platyrhynchos*) induced by AFB1 through regulating Nrf2-ARE and NF-κB signaling pathways. Foods.

[156] El-Sheshtawy S.M., El-Zoghby A.F., Shawky N.A., Samak D.H. (2021). Aflatoxicosis in Pekin duckling and the effects of treatments with lycopene and silymarin. Vet. World.

[157] Ibrahim E.A., Yousef M.I., Ghareeb D.A., Augustyniak M., Giesy J.P., Aboul-Soud M.A.M., El Wakil A. (2022). Artichoke leaf extract-mediated neuroprotection against effects of aflatoxin in male rats. BioMed Res. Int..

[158] Wan F., Tang L., Rao G., Zhong G., Jiang X., Wu S., Huang R., Tang Z., Ruan Z., Chen Z. (2022). Curcumin activates the Nrf2 Pathway to alleviate AFB1-induced immunosuppression in the spleen of ducklings. Toxicon.

[159] Khattab M.S., Aly T.A.A., Mohamed S.M., Naguib A.M.M., Al-Farga A., Abdel-Rahim E.A. (2022). *Hordeum vulgare* L. microgreen mitigates reproductive dysfunction and oxidative stress in streptozotocin-induced diabetes and aflatoxicosis in male rats. Food Sci. Nutr..

[160] Wang L., He K., Wang X., Wang Q., Quan H., Wang P., Xu X. (2022). Recent progress in visual methods for aflatoxin detection. Crit. Rev. Food Sci. Nutr..

[161] Wang Y., Liu F., Liu M., Zhou X., Wang M., Cao K., Jin S., Shan A., Feng X. (2022). Curcumin mitigates aflatoxin B1-induced liver injury via regulating the NLRP3 inflammasome and Nrf2 signaling pathway. Food Chem. Toxicol..

[162] Pauletto M., Giantin M., Tolosi R., Bassan I., Bardhi A., Barbarossa A., Montanucci L., Zaghini A., Dacasto M. (2023). Discovering the protective effects of quercetin on aflatoxin B1-induced toxicity in bovine foetal hepatocyte-derived cells (BFH12). Toxins.

[163] Dazuk V., Tarasconi L., Molosse V.L., Cécere B.G.O., Deolindo G.L., Strapazzon J.V., Bottari N.B., Bissacotti B.F., Schetinger M.R.C., Sareta L. (2023). Can the inclusion of a vegetable biocholine additive in pig feed contaminated with aflatoxin reduce toxicological impacts on animal health and performance?. Animals.

[164] Hassan M.A., Abo-Elmaaty A.M.A., Zaglool A.W., Mohamed S.A.M., Abou-Zeid S.M., Farag M.R., Alagawany M., Di Cerbo A., Azzam M.M., Alhotan R. (2023). *Origanum vulgare* essential oil modulates the AFB1-induced oxidative damages, nephropathy, and altered inflammatory responses in growing rabbits. Toxins.

[165] Altyar A.E., Kensara O.A., Sayed A.A., Aleya L., Almutairi M.H., Zaazouee M.S., Elshanbary A.A., El-Demerdash F.M., Abdel-Daim M.M. (2023). Acute aflatoxin B1-induced hepatic and cardiac oxidative damage in rats: Ameliorative effects of morin. Heliyon.

[166] Velazhahan R., Al-Sadi A.M., Waly M.I., Soundra Pandian S.B., Al-Sabahi J., Al-Farsi K. (2024). Aflatoxin B1 detoxification and antioxidant effect of selected Omani medicinal plants against aflatoxin B1-induced oxidative stress pathogenesis in the mouse liver. Appl. Sci..

[167] Khalid S.A., Elokle A.A. (2025). Evaluation of pumpkin seed oil and its chitosan-Arabic gum nanoparticles on the viability of *Aspergillus flavus* and inhibition of total aflatoxin in beef sausage. Food Control.

[168] Pascale M., Logrieco A.F., Graeber M., Hirschberger M., Reichel M., Lippolis V., De Girolamo A., Lattanzio V.M.T., Slettengren K. (2020). Aflatoxin reduction in maize by industrial-scale cleaning solutions. Toxins.

[169] Sipos P., Peles F., Brassó D.L., Béri B., Pusztahelyi T., Pócsi I., Győri Z. (2021). Physical and chemical methods for reduction in aflatoxin content of feed and food. Toxins.

[170] Rastegar H., Shoeibi S., Yazdanpanah H., Amirahmadi M., Khaneghah A.M., Campagnollo F.B., Sant’Ana A.S. (2017). Removal of aflatoxin B1 by roasting with lemon juice and/or citric acid in contaminated pistachio nuts. Food Control.

[171] Shen M.-H., Singh R.K. (2021). Effect of rotating peanuts on aflatoxin detoxification by ultraviolet C light and irradiation uniformity evaluated by AgCl-based bosimeter. Food Control.

[172] Jubeen F., Sher F., Hazafa A., Zafar F., Ameen M., Rasheed T. (2020). Evaluation and detoxification of aflatoxins in ground and tree nuts using food grade organic acids. Biocatal. Agric. Biotechnol..

[173] Pereyra C.M., Gil S., Cristofolini A., Bonci M., Makita M., Monge M.P., Montenegro M.A., Cavaglieri L.R. (2018). The production of yeast cell wall using an agroindustrial waste influences the wall thickness and is implicated on the aflatoxin B1 adsorption process. Food Res. Int..

[174] Yiannikouris A., Apajalahti J., Siikanen O., Dillon G.P., Moran C.A. (2021). *Saccharomyces cerevisiae* cell wall-based adsorbent reduces aflatoxin B1 absorption in rats. Toxins.

[175] Čolović R., Puvača N., Cheli F., Avantaggiato G., Greco D., Đuragić O., Kos J., Pinotti L. (2019). Decontamination of mycotoxin-contaminated feedstuffs and compound feed. Toxins.

[176] Wang S.-Y., Herrera-Balandrano D.D., Shi X.-C., Chen X., Liu F.-Q., Laborda P. (2023). Occurrence of aflatoxins in water and decontamination strategies: A review. Water Res..

[177] Mata A.T., Ferreira J.P., Oliveira B.R., Batoréu M.C., Barreto Crespo M.T., Pereira V.J., Bronze M.R. (2015). Bottled water: Analysis of mycotoxins by LC–MS/MS. Food Chem..

[178] Oliveira B.R., Mata A.T., Ferreira J.P., Barreto Crespo M.T., Pereira V.J., Bronze M.R. (2018). Production of mycotoxins by filamentous fungi in untreated surface water. Environ. Sci. Pollut. Res..

[179] Picardo M., Sanchís J., Núñez O., Farré M. (2020). Suspect screening of natural toxins in surface and drinking water by high performance liquid chromatography and high-resolution mass spectrometry. Chemosphere.

[180] He K., Quan H., Wang L., Zhang J., Wang H., Zhu X., Xu X. (2023). Engineering cotton fibers with zirconium metal-organic frameworks as a recyclable material for sensing and removing aflatoxins in water. Sens. Actuators B Chem..

[181] Stanley J., Patras A., Pendyala B., Bansode R.R. (2020). Performance of a UV-A LED system for degradation of aflatoxins B1 and M1 in pure water: Kinetics and cytotoxicity study. Sci. Rep..

[182] Chen J., Shi H., Gong M., Chen H., Teng L., Xu P., Wang Y., Hu Z., Zeng Z. (2024). β-Lactoglobulin-based aerogels: Facile preparation and sustainable removal of organic contaminants from water. Int. J. Biol. Macromol..

[183] Siri-Anusornsak W., Kolawole O., Soiklom S., Petchpoung K., Keawkim K., Chuaysrinule C., Maneeboon T. (2024). Innovative use of *Spirogyra* spp. biomass for the sustainable adsorption of aflatoxin B1 and ochratoxin A in aqueous solutions. Molecules.

[184] FAO, OIE, WHO (2019). Taking a Multisectoral One Health Approach: A Tripartite Guide to Addressing Zoonotic Diseases in Countries.

[185] Humboldt-Dachroeden S., Mantovani A. (2021). Assessing Environmental Factors within the One Health Approach. Medicina.

[186] Pettan-Brewer C., Martins A.F., de Abreu D.P.B., Brandão A.P.D., Barbosa D.S., Figueroa D.P., Cediel N., Kahn L.H., Brandespim D.F., Velásquez J.C.C. (2021). From the Approach to the Concept: One Health in Latin America—Experiences and Perspectives in Brazil, Chile, and Colombia. Front. Public Health.

[187] Mor N. (2023). Organising for One Health in a developing country. One Health.

[188] Kumar I., Rawat J., Mohd N., Husain S. (2021). Opportunities of Artificial Intelligence and Machine Learning in the Food Industry. J. Food Qual..

[189] Kim Y.K., Qin J., Baek I., Lee K.M., Kim S.Y., Kim S., Chan D., Herrman T.J., Kim N., Kim M.S. (2023). Detection of aflatoxins in ground maize using a compact and automated Raman spectroscopy system with machine learning. Curr. Res. Food Sci..

[190] Sandlin N., Russell Kish D., Kim J., Zaccaria M., Momeni B. (2022). Current and emerging tools of computational biology to improve the detoxification of mycotoxins. Appl. Environ. Microbiol..

[191] Opoku B., Osekre E.A., Opit G., Bosomtwe A., Bingham G.V. (2023). Evaluation of Hermetic Storage Bags for the Preservation of Yellow Maize in Poultry Farms in Dormaa Ahenkro, Ghana. Insects.

[192] Kumar D., Kalita P. (2017). Reducing postharvest losses during storage of grain crops to strengthen food security in developing countries. Foods.

[193] Drago E., Campardelli R., Pettinato M., Perego P. (2020). Innovations in smart packaging concepts for food: An extensive review. Foods.

[194] Moreno M.A., Vallejo A.M., Ballester A.R., Zampini C., Isla M.I., López-Rubio A., Fabra M.J. (2020). Antifungal edible coatings containing Argentinian propolis extract and their application in raspberries. Food Hydrocoll..

[195] Escamilla-García M., Calderón-Domínguez G., Chanona-Pérez J.J., Mendoza-Madrigal A.G., Di Pierro P., García-Almendárez B.E., Amaro-Reyes A., Regalado-González C. (2017). Physical, structural, barrier, and antifungal characterization of chitosan-zein edible films with added essential oils. Int. J. Mol. Sci..

[196] Luz C., Calpe J., Saladino F., Luciano F.B., Fernandez-Franzon M., Manes J., Meca G. (2018). Antimicrobial packaging based on E-polylysine bioactive film for the control of mycotoxigenic fungi in vitro and in bread. J. Food Process. Preserv..

[197] Kazemian-Bazkiaee F., Ebrahimi A., Hosseini S.M., Shojaee-Aliabadi S., Farhoodi M., Rahmatzadeh B., Sheikhi Z. (2020). Evaluating the protective effect of edible coatings on lipid oxidation, fatty acid composition, and aflatoxins levels of roasted peanut kernels. Food Meas..

[198] El-Sayed H.S., El-Sayed S.M., Mabrouk A.M.M., Nawwar G.A., Youssef A.M. (2021). Development of eco-friendly probiotic edible coatings based on chitosan, alginate and carboxymethyl cellulose for improving the shelf life of UF soft cheese. J. Polym. Environ..

[199] Gomes A.S.d.L.P.B., Weber S.H., Luciano F.B. (2024). Resistance of transgenic maize cultivars to mycotoxin production—systematic review and meta-analysis. Toxins.

[200] Rajasekaran K., Sayler R.J., Sickler C.M., Majumdar R., Jaynes J.M., Cary J.W. (2018). Control of *Aspergillus flavus* growth and aflatoxin production in transgenic maize kernels expressing a tachyplesin-derived synthetic peptide, AGM182. Plant Sci..

[201] Thakare D., Zhang J., Wing R.A., Cotty P.J., Schmidt M.A. (2017). Aflatoxin-free transgenic maize using host-induced gene silencing. Sci. Adv..

[202] Raruang Y., Omolehin O., Hu D., Wei Q., Promyou S., Parekattil L.J., Rajasekaran K., Cary J.W., Wang K., Chen Z.-Y. (2023). Targeting the *Aspergillus flavus p2c* gene through host-induced gene silencing reduces *A. flavus* infection and aflatoxin contamination in transgenic maize. Front. Plant Sci..

